# Global Analysis of the Fungal Microbiome in Cystic Fibrosis Patients Reveals Loss of Function of the Transcriptional Repressor Nrg1 as a Mechanism of Pathogen Adaptation

**DOI:** 10.1371/journal.ppat.1005308

**Published:** 2015-11-20

**Authors:** Sang Hu Kim, Shawn T. Clark, Anuradha Surendra, Julia K. Copeland, Pauline W. Wang, Ron Ammar, Cathy Collins, D. Elizabeth Tullis, Corey Nislow, David M. Hwang, David S. Guttman, Leah E. Cowen

**Affiliations:** 1 Department of Molecular Genetics, University of Toronto, Toronto, Ontario, Canada; 2 Department of Laboratory Medicine and Pathobiology, University of Toronto, Toronto, Ontario, Canada; 3 Centre for the Analysis of Genome Evolution & Function, University of Toronto, Toronto, Ontario, Canada; 4 Department of Cell & Systems Biology, University of Toronto, Toronto, Ontario, Canada; 5 Donnelly Centre for Cellular and Biomolecular Research, University of Toronto, Toronto, Ontario, Canada; 6 Division of Respirology, St. Michael’s Hospital, Toronto, Ontario, Canada; 7 Faculty of Pharmaceutical Sciences, The University of British Columbia, Vancouver, British Columbia, Canada; Geisel School of Medicine at Dartmouth, UNITED STATES

## Abstract

The microbiome shapes diverse facets of human biology and disease, with the importance of fungi only beginning to be appreciated. Microbial communities infiltrate diverse anatomical sites as with the respiratory tract of healthy humans and those with diseases such as cystic fibrosis, where chronic colonization and infection lead to clinical decline. Although fungi are frequently recovered from cystic fibrosis patient sputum samples and have been associated with deterioration of lung function, understanding of species and population dynamics remains in its infancy. Here, we coupled high-throughput sequencing of the ribosomal RNA internal transcribed spacer 1 (ITS1) with phenotypic and genotypic analyses of fungi from 89 sputum samples from 28 cystic fibrosis patients. Fungal communities defined by sequencing were concordant with those defined by culture-based analyses of 1,603 isolates from the same samples. Different patients harbored distinct fungal communities. There were detectable trends, however, including colonization with *Candida* and *Aspergillus* species, which was not perturbed by clinical exacerbation or treatment. We identified considerable inter- and intra-species phenotypic variation in traits important for host adaptation, including antifungal drug resistance and morphogenesis. While variation in drug resistance was largely between species, striking variation in morphogenesis emerged within *Candida* species. Filamentation was uncoupled from inducing cues in 28 *Candida* isolates recovered from six patients. The filamentous isolates were resistant to the filamentation-repressive effects of *Pseudomonas aeruginosa*, implicating inter-kingdom interactions as the selective force. Genome sequencing revealed that all but one of the filamentous isolates harbored mutations in the transcriptional repressor *NRG1*; such mutations were necessary and sufficient for the filamentous phenotype. Six independent *nrg1* mutations arose in *Candida* isolates from different patients, providing a poignant example of parallel evolution. Together, this combined clinical-genomic approach provides a high-resolution portrait of the fungal microbiome of cystic fibrosis patient lungs and identifies a genetic basis of pathogen adaptation.

## Author Summary

Microbial cells vastly outnumber human cells in our bodies, yet we are only beginning to understand how these microbes influence human health and disease. One disease for which microbial communities are especially important is cystic fibrosis, where persistent lung infections can be lethal. Fungi are associated with poor respiratory function, but how fungal communities change with disease progression or treatment remains enigmatic. Here, we assess the dynamics of fungal communities by combining high-throughput sequencing of sputum samples from 28 patients with detailed analysis of phenotypes and genotypes of 1,603 fungal isolates. We found stable communities dominated by *Candida* and *Aspergillus*, and diversity in traits important for host adaptation. Antifungal drug resistance varied largely between species, while morphogenesis varied within species. For *Candida* species, the capacity to transition between yeast and filaments is a key virulence trait that is normally regulated by inducing cues, however, 28 isolates grew as filaments without such cues. Filamentation was due to loss-of-function mutations in the transcriptional regulator *NRG1* in most isolates, which conferred resistance to the filament-repressive effects of a common bacterial pathogen. This work provides a portrait of the fungal microbiome associated with a lethal disease, and illuminates a genetic basis of pathogen adaptation.

## Introduction

The microbiome has a profound impact on diverse facets of human biology and disease. Anatomical sites such as the respiratory tract that were once thought to be sterile are now recognized to harbor complex microbial communities in healthy individuals as well as those suffering from a multitude of conditions [1]. One respiratory condition for which chronic and complex polymicrobial communities are now appreciated to have a severe impact on prognosis is cystic fibrosis. Patients with this genetic disorder caused by reduced function of the transmembrane conductance regulator CFTR experience thick mucus accumulation in airways, which renders them vulnerable to chronic airway infection and repeated episodes of pulmonary exacerbation [2]. Our understanding of microbiomes of the lung and respiratory tract of cystic fibrosis patients is based largely on studies focused on bacteria [2,3]. Traditionally, opportunistic bacterial pathogens such as *Pseudomonas aeruginosa*, *Burkholderia* species, and *Staphylococcus aureus* have been implicated in pulmonary exacerbations [3]. Molecular approaches have provided additional insight into culprits of respiratory infections in cystic fibrosis patients, as with the implication of a bacterial opportunistic pathogen of the *Streptococcus milleri* group that had been previously overlooked [2,4]. The resultant episodes of pulmonary exacerbations cause decline in lung function, ultimately leading to patient death.

There is a growing appreciation of the importance of fungi in the lung microbiome. With an excess of 50,000 fungal spores/m^3^ of air in some seasons, the human respiratory tract is exposed to vast quantities of fungi [1]. The lung is often the initial site of colonization from which dissemination can lead to systemic fungal infections, especially in immunocompromised individuals and those treated with antibacterial agents [5]. Fungi are frequently isolated from cystic fibrosis patient sputum samples, where *Candida albicans* and *Aspergillus fumigatus* are the most prevalent species, identified in 40–70% of patients [6,7]. Fungi can have a profound impact on cystic fibrosis patients by inducing local host inflammatory responses as well as causing direct damage of the respiratory mucosa, thereby contributing to progressive deterioration of lung function. For example, allergic bronchopulmonary aspergillosis (ABPA) has been reported in up to 10.9% of cystic fibrosis patients, while chronic *A*. *fumigatus* infection leads to complications such as asthma, bronchitis, aspergilloma, and invasive pulmonary infection after lung transplants [8,9]. Although *C*. *albicans* is frequently isolated from cystic fibrosis patient sputum samples, the clinical impact is not yet clear. *C*. *albicans* has been reported as the leading cause of allergic bronchopulmonary mycosis (ABPM) after *Aspergillus* species in the general population [10], and chronic *Candida* infection has been associated with increased pulmonary exacerbations in cystic fibrosis patients [11]. The few microbiome studies that have focused on fungi to date have revealed that communities in the lungs of healthy people are dominated by fungal species that are ubiquitous in the environment [12], while communities in cystic fibrosis patients are dominated by species of *Candida*, *Aspergillus*, and *Malassezia* [7,13]. Analysis of fungal microbiomes via high-throughput sequencing provides a powerful approach to monitor changes in species dynamics over the course of clinical exacerbations and treatments.

Leveraging phenotypic and genotypic characterization of microbial populations in the host provides an important complement to monitoring species level dynamics that is key for understanding microbial ecology and evolution in context. Fungal adaptation in the host has been studied most extensively in *Candida* species, where genome sequencing has been used to elucidate the genetic basis of the emergence of drug resistance in pathogen populations over the course of patient treatment [14–16]. For *C*. *albicans*, a natural member of the human mucosal microbiota and an important opportunistic pathogen, another key trait for which variation has been observed among clinical isolates is morphogenesis [16]. *C*. *albicans* can transition between yeast and filamentous morphologies in response to specific environmental cues, which has a profound impact on virulence and host adaptation [17]. *C*. *albicans* morphology can also be modulated by inter-kingdom interactions with bacterial pathogens commonly associated with cystic fibrosis. *P*. *aeruginosa* inhibits *C*. *albicans* filamentation by secreting molecules such as pyocyanin and 3-oxo-C12 homoserine lactone, and selectively kills *C*. *albicans* filaments by forming biofilms on their surfaces [18–20]. *Burkholderia cenocepacia* is also able to inhibit *C*. *albicans* filamentation by secreting cis-2-dodecenoic acid [21]. In contrast, *S*. *aureus* has been reported to engage in mutualistic interactions with *C*. *albicans*, where it preferentially attaches to *C*. *albicans* cells in mixed biofilms and enhances disease severity in a mouse co-infection model [22,23]. Thus, cystic fibrosis patient lungs provide a clinically important and powerful context for tracking the dynamics and evolution of fungal communities and populations in the host.

In this study, we combined high-throughput sequencing of the ribosomal RNA internal transcribed spacer 1 (ITS1) with phenotypic and genotypic analyses to provide the most comprehensive assessment of the cystic fibrosis lung fungal microbiome (mycobiome) to date. We found that the fungal communities identified by culture-independent sequencing of 89 sputum samples from 28 cystic fibrosis patients were concordant with those defined by culture-based analyses of 1,603 isolates from the same samples. Both methods identified *C*. *albicans* as the dominant fungus from most cystic fibrosis patients, although some patients were primarily colonized by *Candida parapsilosis*, *A*. *fumigatus*, or a mixture of different fungal species including *Aspergillus flavus*, *Aspergillus terreus*, *Candida glabrata*, and *Candida tropicalis*. Our phenotypic characterization revealed extensive inter-species and intra-species diversity in growth characteristics and antifungal drug resistance. For *C*. *albicans*, we recovered multiple isolates from different patients over time that displayed filamentous growth in the absence of any inducing cue. Whole genome sequencing identified loss-of-function mutations in *NRG1* as the genetic basis of the filamentous phenotype. Mutations in the transcriptional repressor *NRG1* were identified in 24 out of 25 filamentous *C*. *albicans* isolates that were recovered from six different patients, suggesting that this is a common mechanism of adaptation. The filamentous *C*. *albicans* clinical isolates were resistant to the filamentation-repressive effects of the dominant bacterial pathogens present in the patients from which they were recovered, including *P*. *aeruginosa* and *Burkholderia multivorans*, as well as a structural analog of the quorum sensing molecule pyocyanin. Thus, we provide a high-resolution portrait of species and population level dynamics in the fungal microbiome of cystic fibrosis patient lungs, and identify the genetic basis of pathogen adaptation in the host.

## Results

### Sputum sampling and patient characteristics

We collected a total of 111 sputum samples from 28 adult CF patients from St. Michael’s hospital in Toronto. Of the 28 participants, 20 patients provided at least 2 sputum samples between 3 and 56 months apart for longitudinal analyses (S1 Table). Patients CF020, CF025, CF027, CF060, CF098, and CF107 were diagnosed with ABPA, and patients CF014, CF025, CF028, CF060, CF098, and CF107 were prescribed fluconazole, itraconazole, or posaconazole during sampling period (S1 Table). Other patients were prescribed antifungal drugs prior or after the sputum collection periods, including CF006 (nystatin), CF011 (nystatin), CF020 (nystatin), and CF027 (fluconazole). According to the clinical microbiology data, *P*. *aeruginosa* was the most commonly isolated microorganism from the sputum samples, followed by *Aspergillus* species, *Stenotrophomonas maltophila*, and *B*. *cepacia*, among other bacterial and fungal species (S1 Table).

### Characterization of the cystic fibrosis mycobiome utilizing high-throughput ITS1 sequencing of sputum DNA samples

**Fig 1:**
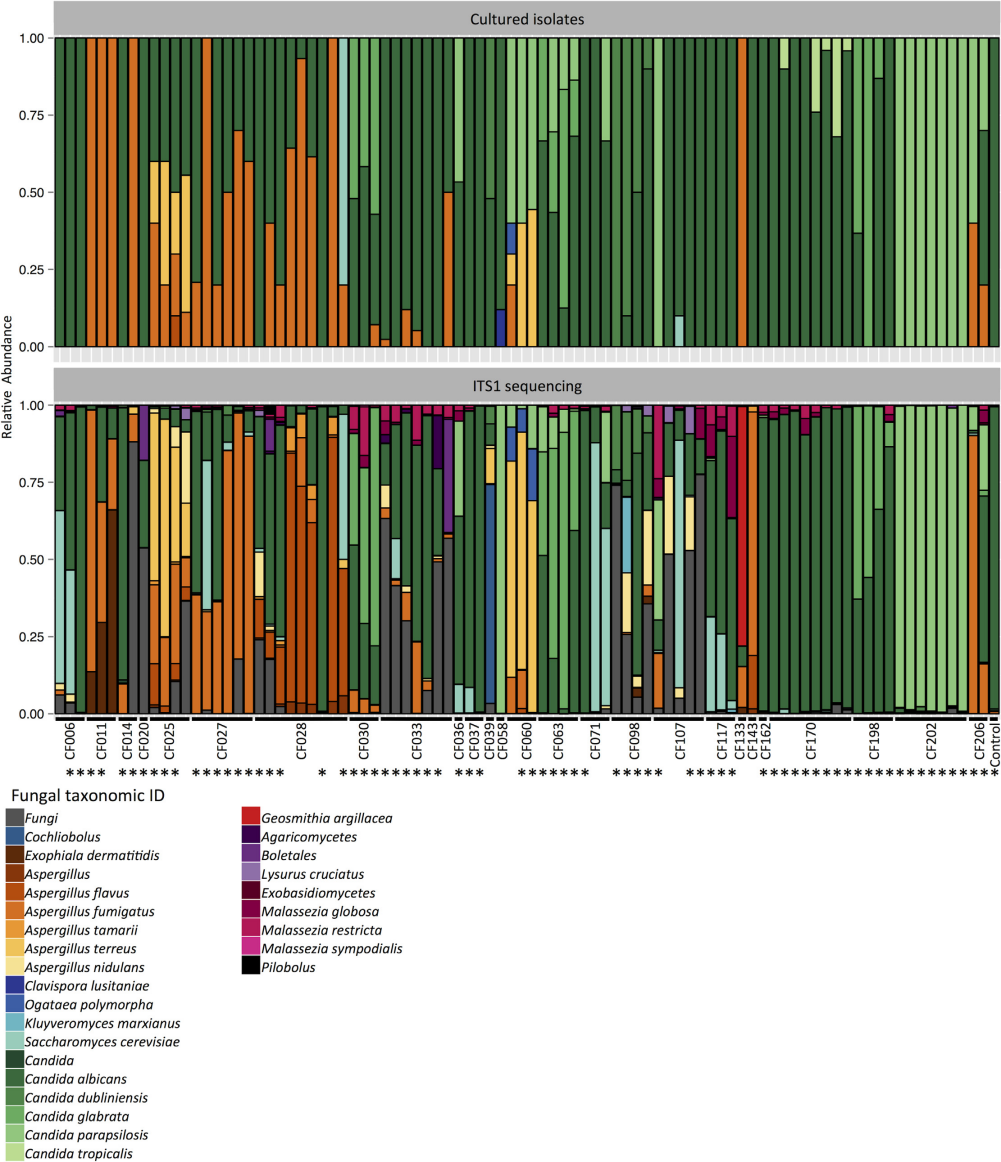
Portrait of the cystic fibrosis lung mycobiome. Relative abundance of fungal species in 89 sputum samples from 28 patients, identified by culture (top) and ITS1 sequencing (bottom). *: Pearson correlation > 0.5.

We used a molecular approach to characterize the cystic fibrosis mycobiome, and extracted DNA from 111 sputum samples for amplification and high-throughput sequencing of ITS1, which is a commonly used locus for fungal species identification [13]. We were able to amplify ITS1 from 89 of the 111 sputum DNA samples, although all of the 111 sputum samples produced viable fungal isolates. Amplification of ITS1 did not track with the number of fungal isolates from the corresponding sputum sample (S1 Table and S1 File). The amplified products were barcoded, pooled, and sequenced using Illumina short-read sequencing methods, and the taxonomic identities were assigned to each read. In most sputum samples, *Candida* species were the most abundant fungi (51 samples out of the 89), followed by *Aspergillus* species (18 samples) (Fig 1, and S1 Fig). We detected pathogens previously reported in other cystic fibrosis studies, such as *Exophiala dermatitidis* [24] from CF011, *Geosmithia argillacea* [25] from CF133, and *Malassezia* species [7,13] from CF107 and CF117 (Fig 1). Furthermore, we detected opportunistic fungal pathogens yet to be reported in the context of cystic fibrosis to date, such as *Cochliobolus* species (anamorphs *Curvularia*) [26] from CF039 and *Kluyveromyces marxianus* (anamorph *Candida kefyr*) [27] from CF098 (Fig 1). The fact that we identified additional species in our sputum samples may reflect the more extensive sampling in our study design.

Next, we tested for associations between taxonomic abundance and patient characteristics including clinical state, lung disease stage, *Pseudomonas* load, triazole therapy, lung function index FEV_1_% predicted value (based on the volume exhaled during the first second of forced expiration for a population average of similar age), and body mass index (BMI), as described in the patient clinical data (S1 Table). We utilized the Simpson’s diversity index, which measures community diversity within the samples and produces a numerical output between 0 to 1, where 0 indicates no diversity and 1 indicates maximum diversity. There was no significant association between the Simpson’s diversity index from individual samples and patient characteristics, as was the case with Shannon diversity index (S2–S4 Figs). Based on a previous report that chronic *C*. *albicans* colonization leads to reduced FEV_1_ values in cystic fibrosis patients [11], we also compared the correlation between *C*. *albicans* read counts and patient FEV_1_% predicted values, which revealed a weak, yet significant correlation (r = -0.14, S5 Fig). In order to compare the differences in taxonomic compositions between the sputum samples over time, we selected sputum samples from patients with more than 1 sampling points (19 patients) and utilized the Bray-Curtis dissimilarity index. This measures the dissimilarity between samples and produces a numerical output between 0 to 1, where 0 indicates no dissimilarity and 1 indicates no similarity. We did not identify clear community structures based on patient status, but most samples clustered based on patient ID (S6 and S7 Figs). The changes observed in taxonomic composition (Fig 1) or Bray-Curtis dissimilarity index (S6 Fig) did not correlate with the changes in patient status (S1 Table).

### Species identification and phenotypic characterization of fungal isolates from CF patient sputum samples

We recovered fungal isolates from sputum samples by plating homogenized sputum onto medium containing antibiotics to inhibit bacterial growth (S1 File). We sampled up to 50 colonies per sputum sample, with a mean of 14 colonies and a range of 1 to 50 colonies. For sputum samples with more than 50 fungal colonies, an effort was made to maintain the relative abundance of colony morphologies during sampling. Fungal isolates were initially grouped into two major categories: 182 mold isolates from 10 patients and 1,421 yeast isolates from 26 patients (S1 File). In order to assess phenotypic diversity and define phenotypic classes to facilitate species assignments by sequencing, all isolates were then screened for growth rate, antifungal susceptibility, and cellular morphology. For molds, conidia coloration was noted, and yeast isolates were plated on CHROMagar Candida [28]. A minimum of two representative isolates of each phenotypic class were assigned to species by sequencing the internal transcribed spacer 2 (ITS2) region of the ribosomal RNA, which is also commonly used for fungal species identification [29]. In all cases, sequencing confirmed that isolates with concordant phenotypic profiles were the same species.

Different patients harbored distinct species of molds. All 182 mold isolates were classified into three species: *A*. *fumigatus*, *A*. *flavus*, and *A*. *terreus* (Fig 1). Since the ITS2 locus is not able to discriminate between closely related species within Fumigati, which includes *A*. *fumigatus* [30], we selected a minimum of two representative isolates initially identified as *A*. *fumigatus* based on ITS2 sequence per patient, and sequenced the β-tubulin locus to confirm the species identity. We recovered 103 *A*. *fumigatus* isolates from nine patients, and it was the most frequently recovered mold from the sputum samples. We recovered *A*. *flavus* isolates from two patients, with 56 of the isolates from a single patient (CF028) and one from another patient. We also recovered 22 *A*. *terreus* isolates from two patients (Fig 1).

As with the molds, different patients harbored distinct species of yeasts. *C*. *albicans* was the most frequent species recovered, with 1,056 isolates from 23 patients (Fig 1). Furthermore, *C*. *albicans* was the dominant fungal species in 16 of the 23 patients. We recovered 162 *C*. *parapsilosis* isolates from nine patients and it was the dominant fungal species in four of the nine patients. We recovered 129 *C*. *glabrata* isolates from three patients and it was the dominant fungal species in two of the three patients. We also recovered 24 *C*. *tropicalis* isolates from a single patient (Fig 1).

We compared the relative abundances of different fungal species identified from the cultured isolates to the relative abundances of ITS1 reads from the corresponding samples and found that the results were high correlated at both species and genus level (Fig 1 and S1 Fig, Pearson correlation 0.70 and 0.78, respectively). However, we were not able to culture some fungi that showed greater than 10% relative abundances based on ITS1 sequencing of specific sputum samples, including *Cochliobolus* species, *E*. *dermatitidis*, *A*. *nidulans*, *K*. *marxianus*, *G*. *argillacea*, and *Malassezia* species, many of which require specific growth conditions that were not utilized in our study (Fig 1) [31,32]. ITS1 sequencing also had limitations relative to the culture-based approach as it did not detect *Clavispora lusitaniae* (two isolates out of 25 from CF058) and *C*. *tropicalis* (24 out of 254 from CF170) (S1 File).

### Intra-Species and Inter-Species Variation in Antifungal Susceptibility

**Fig 2:**
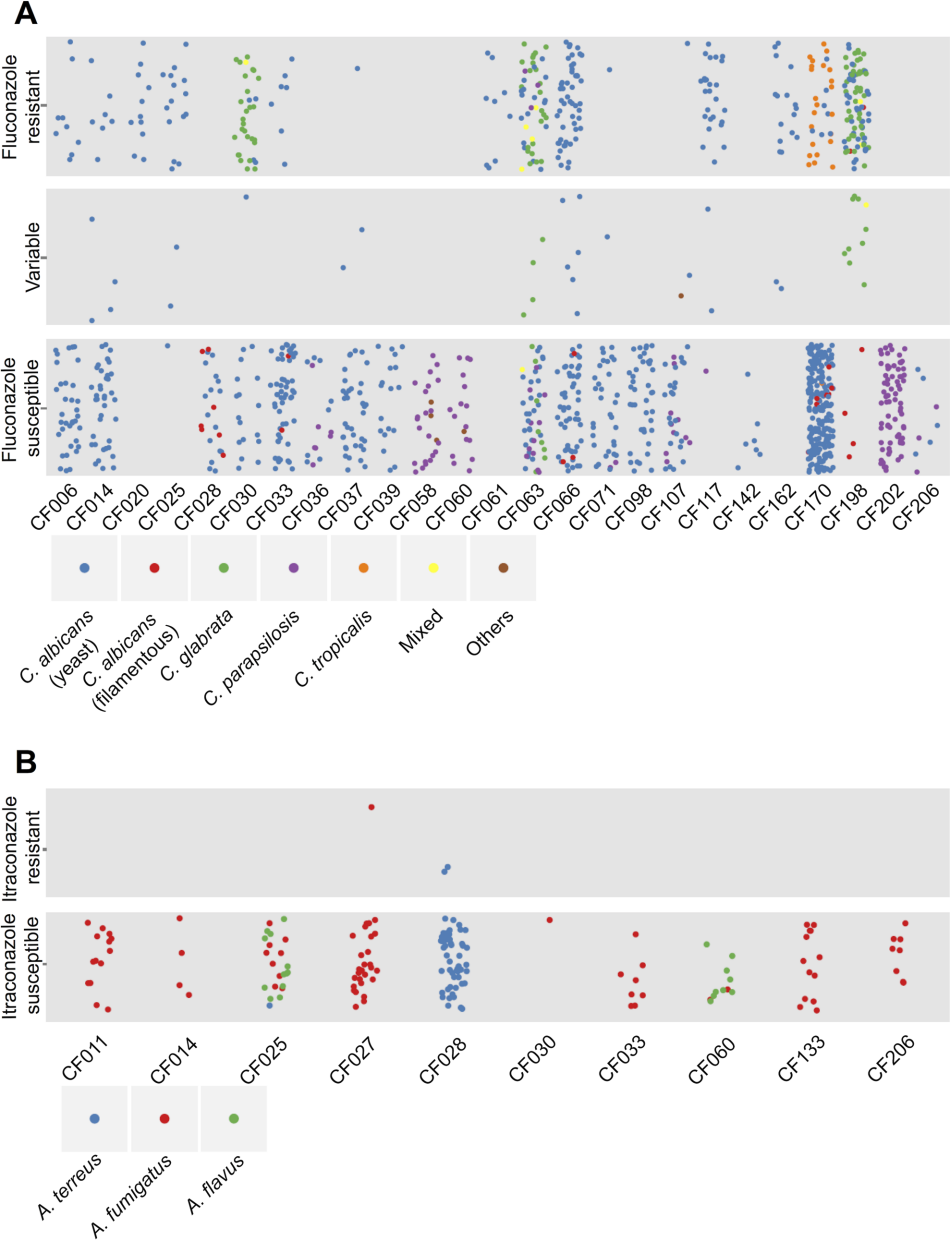
Diversity of species and antifungal resistance profiles of 1,603 fungal isolates from cystic fibrosis patients. Each dot represents an isolate from the corresponding cystic fibrosis patient and colours represent species identity. The vertical dispersion of isolates within each phenotypic class is simply for visual clarity, as with the horizontal dispersion of isolates from an individual patient. (A) Summary of yeast isolates. The isolates are categorized as: “Fluconazole resistant” if their relative growth with fixed concentration of fluconazole at 128 μg/ml was greater than 2 times that of the relative growth of reference *C*. *albicans* strain SN95; “Fluconazole susceptible” if their relative growth was less than 2 times the relative growth of SN95; and “Variable” if their resistance profiles were variable over biological and technical duplicates. *C*. *albicans* is divided into two groups: isolates that show standard yeast morphology in rich medium at 30°C are in blue; and isolates that show filamentous growth under these conditions are in red. (B) Summary of mold isolates. The isolates are categorized as: “Itraconazole resistant” if their relative growth with fixed concentration of itraconazole at 0.5 μg/ml was greater than 2 times that of the relative growth of reference *A*. *fumigatus* strain AF293; and “Itraconazole susceptible” if their relative growth was less than 2 times the relative growth of AF293

We measured the fluconazole susceptibility of the full set of yeast isolates and the itraconazole susceptibility of the 182 mold isolates. Of the 1,421 yeast isolates from 26 patients, 362 isolates from 16 patients showed resistance to a high concentration of fluconazole (128 μg/ml); 20 of these resistant isolates were recovered from three of the eight patients who were treated with azoles, but there was no correlation between the recovery of resistant isolates and prior azole treatment (Fig 2A, S8 Fig, S1 Table, and S1 File) The majority of *C*. *glabrata* and *C*. *tropicalis* isolates were resistant to fluconazole, and most *C*. *parapsilosis* isolates were susceptible (S1 File). Of the 182 mold isolates from 10 patients, most were susceptible to a fixed concentration of itraconazole (0.5 μg/ml). Furthermore, all *A*. *fumigatus* isolates and most *A*. *flavus* isolates were susceptible to a fixed concentration of amphotericin B, but most *A*. *terreus* isolates were resistant (Fig 2B and S9 Fig).

### Variation in Growth Profiles

We examined the growth profiles of the 1,421 yeast and 182 mold isolates in liquid medium by measuring changes in OD_595_ over time and calculating area under the curve as the output of growth. When growth of the yeast isolates was compared to the reference *C*. *albicans* strain SN95, most showed similar profiles except for *C*. *parapsilosis* isolates, which consistently grew less than other isolates (S1 File). Furthermore, we identified some *C*. *albicans* isolates that showed aberrant growth kinetics that was attributable to filamentous growth (S10 Fig, CF170-P2C11 and S1 File). When growth of the mold isolates was compared to the reference *A*. *fumigatus* strain Af293, most showed consistently enhanced growth relative to the reference strain (S11 Fig and S1 File).

### Intra-Species and Inter-Species Variation in Morphology

We examined several additional phenotypes for the mold and yeast species. For the molds we monitored color of conidia to support species assignments. For the yeasts, we monitored cellular morphology of the isolates that showed aberrant growth in rich liquid medium at 30°C, and identified a striking example of intra-species variation in morphology (S10 Fig CF170-P2C11 and S1 File). Of the 1,056 *C*. *albicans* isolates, 25 isolates from five patients exhibited filamentous growth at 30°C in rich medium (Fig 2A, S1 File): in CF028, seven out of the 66 *C*. *albicans* isolates were filamentous; in CF033, two out of 172 *C*. *albicans* isolates were filamentous; in CF066, three out of 114 *C*. *albicans* isolates were filamentous; in CF170, seven out of 229 *C*. *albicans* isolates were filamentous; and in CF198, six out of 43 *C*. *albicans* isolates were filamentous (Fig 2A). We also identified three *C*. *parapsilosis* isolates from patient CF107 showed filamentous growth at 30°C in rich medium (S12A Fig).

### Genome sequence analysis identifies the genetic basis of filamentation in clinical *Candida* isolates

**Fig 3:**
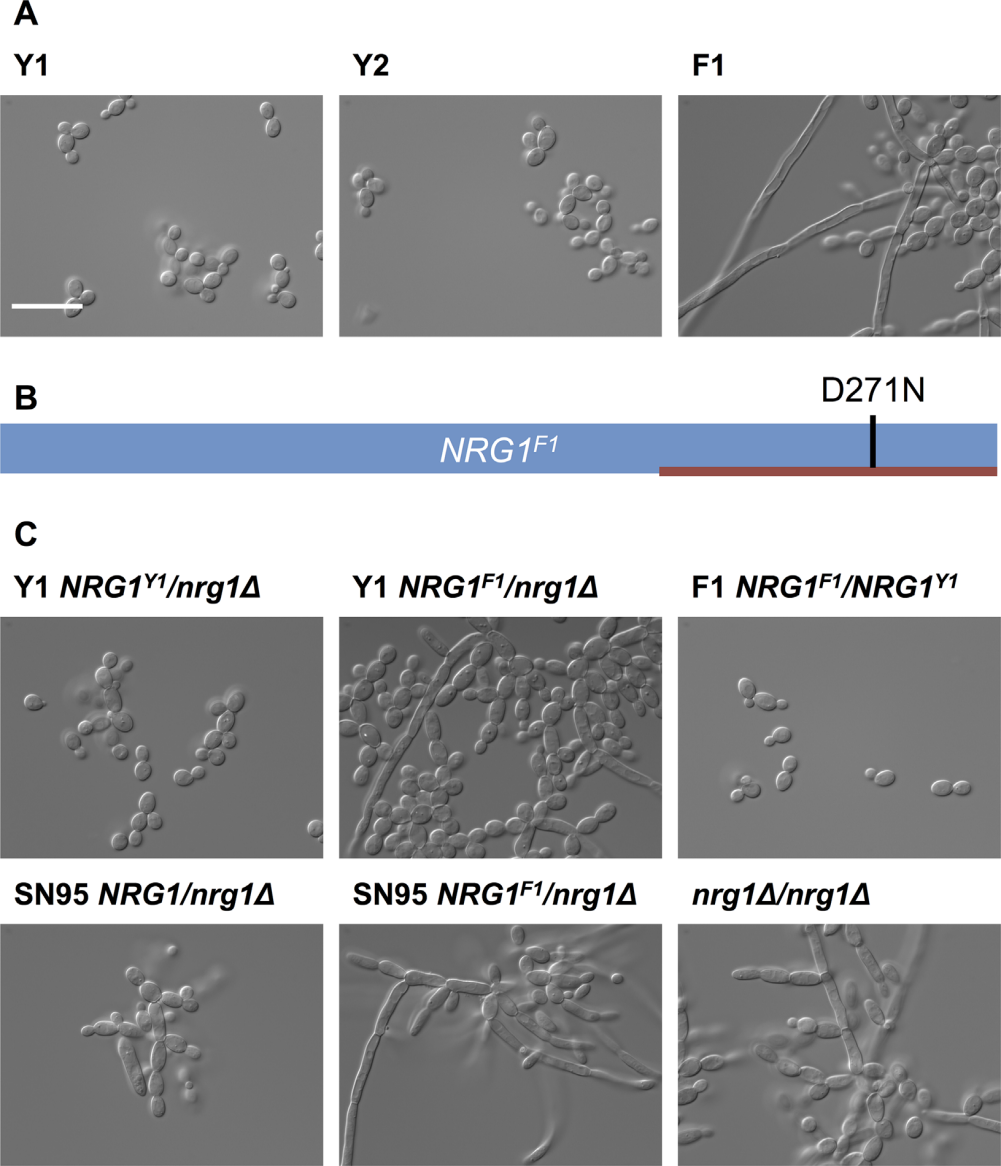
Filamentous growth of *C*. *albicans* clinical isolates is caused by non-synonymous mutation in *NRG1*. (A) DIC images of *C*. *albicans* isolates from patient CF170 cultured in YPD at 30°C. *C*. *albicans* isolates Y1 and Y2 grow in the yeast form, while the filamentous isolate F1 grow as a mixture of yeast and filaments. Scale bar represents 20 μm. (B) Schematic representation of *NRG1* ^*F1*^, showing the homozygous D271N mutation in the C_2_H_2_ zinc finger DNA binding domain (red bar). (C) Functional validation that *NRG1* ^*F1*^ confers filamentous growth. Deletion of one allele of *NRG1* in Y1 (Y1 *NRG1* ^*Y1*^ */nrg1Δ*) does not alter yeast growth morphology, while replacement of the remaining *NRG1* allele in this background with the F1 *NRG1* allele (Y1 *NRG1* ^*F1*^ */nrg1Δ*) causes a filamentation phenotype comparable to that observed in F1. This was also true in an independent laboratory strain, SN95 [38]. Replacing one *NRG1* allele of F1 with the Y1 allele restores yeast form growth. Finally, a homozygous *NRG1* deletion mutant (*nrg1Δ/nrg1Δ*) in an independent background [39] also has comparable growth morphology to F1.

Given that the morphological transition between yeast and filamentous growth is normally a tightly controlled developmental program that profoundly impacts on host adaptation and virulence [33,34], we asked if there was a genetic basis for the altered regulation of this program in the isolates that showed a filamentous phenotype in the absence of any inducing cue. We performed whole-genome sequencing of three *C*. *albicans* isolates from patient CF170, one with the filamentous phenotype under standard conditions, designated F1, and two that grew as yeast, designated Y1 and Y2 (Fig 3A). Sequence reads were aligned to the published *C*. *albicans* genome (SC5314, assembly 21, with the mean depth of coverage being 50X for all assembled sequences) [35]. Unique single nucleotide variants present in F1 were identified by comparing the F1 genome assembly to the assemblies of Y1 and Y2 genomes using MuTect [36] (S2 Table). We identified 76 high-confidence single nucleotide variants in F1: 37 were in non-coding regions; 20 were in coding regions and resulted in synonymous heterozygous mutations; 18 were in coding regions and resulted in non-synonymous heterozygous mutations; and one variant manifested as a homozygous non-synonymous mutation in *NRG1* [37]. This last mutation is compelling, as *NRG1* encodes a transcription factor that is known to repress filamentation (S2 Table). The mutation (D271N) was located within the C_2_H_2_ zinc finger domain of *NRG1* [37], potentially impairing the ability of this transcriptional repressor of filamentation to bind DNA (Fig 3B).

Next, we functionally validated that the mutation in *NRG1* conferred the filamentous phenotype. Since we anticipated that the D271N allele would confer a loss of function and would thus be recessive, we deleted one allele of *NRG1* in Y1 to generate the Y1 *NRG1* ^*Y1*^ */nrg1Δ* strain; this Y1 *NRG1* ^*Y1*^ */nrg1Δ* strain had the same yeast growth morphology as Y1 (Fig 3C). We then replaced the remaining *NRG1* ^*Y1*^ allele with the D271N allele to generate the Y1 *NRG1* ^*F1*^ */nrg1Δ* strain; this Y1 *NRG1* ^*F1*^ */nrg1Δ* strain had a filamentous growth phenotype akin to that observed in F1. This demonstrates that the *NRG1* allele of F1 is sufficient to confer the filamentation phenotype on Y1. To further confirm that the *NRG1* D271 allele is necessary for the filamentation phenotype of F1, we replaced one allele of *NRG1* in strain F1 with the allele from Y1 to generate the F1 *NRG1* ^*F1*^ */NRG1* ^*Y1*^ strain; this F1 *NRG1* ^*F1*^ */NRG1* ^*Y1*^ strain lost the filamentation phenotype, confirming that the *NRG1* mutation is recessive and that it is necessary for the filamentation phenotype of F1. The phenotype of the strains harboring only the F1 allele of *NRG1* was comparable to that of an *nrg1Δ/nrg1Δ* homozygous deletion mutant and an *NRG1* ^*F1*^/*nrg1Δ* mutant in independent laboratory strain background (Fig 3C), consistent with the model that the *NRG1* mutation identified in F1 causes loss of function of this transcriptional repressor of filamentous growth.

**Fig 4:**
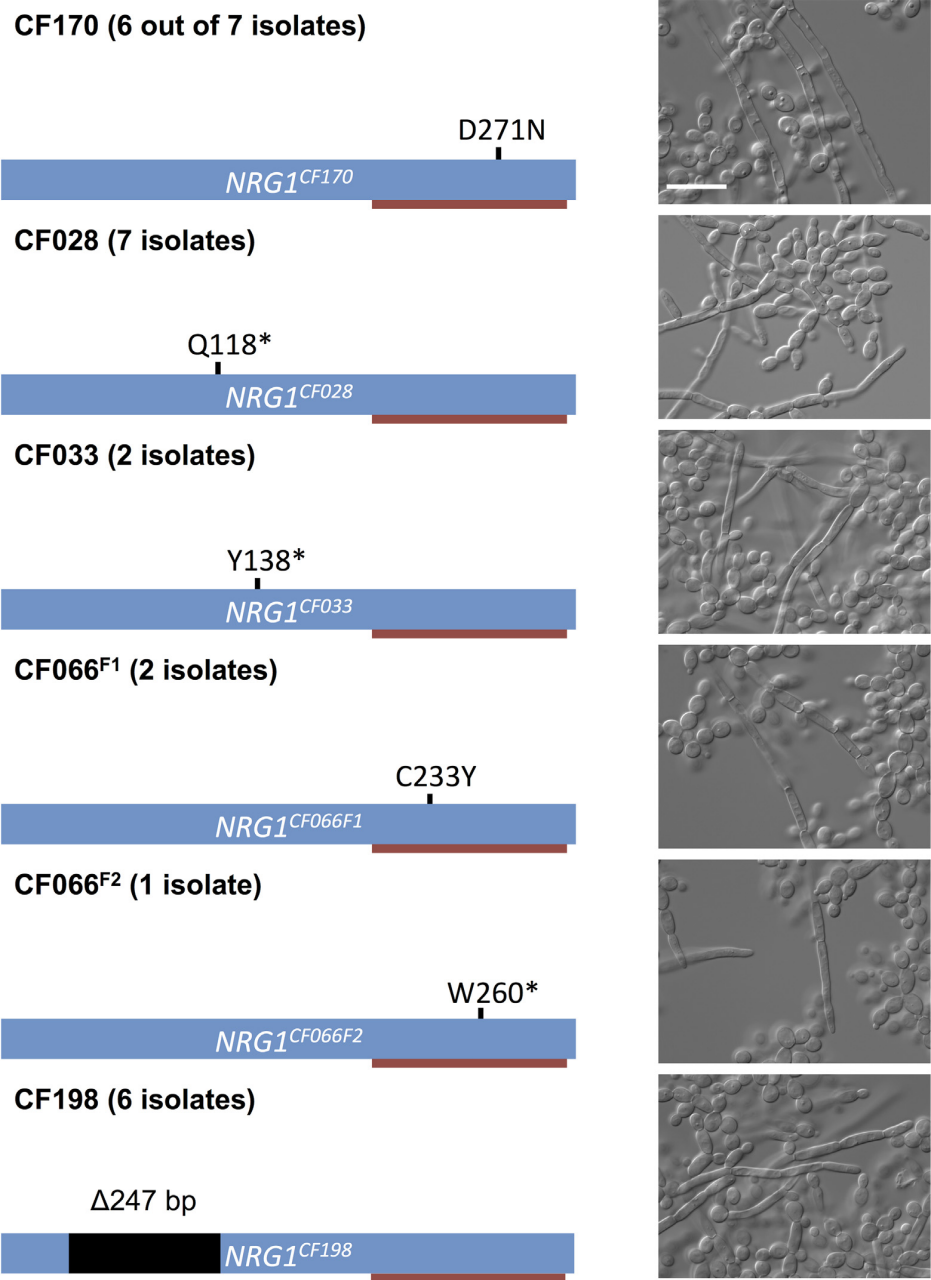
Prevalence of *NRG1* mutations in filamentous *C*. *albicans* isolates from multiple patients. Schematic representation of *NRG1*, the C_2_H_2_ zinc finger DNA binding domain (red bar), and the mutations found in different filamentous isolates and the corresponding microscopy images of representative isolates. Scale bar represents 20 μm.

To determine if the phenotypes of the remaining 24 filamentous *C*. *albicans* isolates that were recovered from five patients were also associated with mutations in *NRG1*, we sequenced *NRG1* from these isolates. We identified five different homozygous mutations unique to the filamentous isolates (Fig 4). From patient CF170, we recovered six additional filamentous *C*. *albicans* isolates; five of these six isolates had homozygous D271N mutations, suggesting persistence of this genotype in the patient. The remaining filamentous isolate (F2) did not have a mutation in *NRG1* or in the surrounding regions, and whole genome sequencing did not reveal any homozygous mutations or strong candidate mutations that would confer the filamentous phenotype (S3 Table). The seven filamentous isolates from patient CF028 each had a homozygous Q118* nonsense mutation in *NRG1*, and both filamentous isolates from CF033 had a homozygous Y138* nonsense mutation in *NRG1*. Two different mutations were identified from the three filamentous isolates recovered from patient CF066, two with a homozygous C233Y mutations in *NRG1*, which is within the C_2_H_2_ zinc finger domain, and one with a homozygous W260* mutation in *NRG1*. Finally, all six filamentous isolates from patient CF198 had a homozygous 247 bp deletion in *NRG1*, leading to a frame shift and introduction of a premature stop codon (Fig 4). Since all of the observed mutations in *NRG1* were located before or within its C_2_H_2_ zinc finger domain, the filamentous growth phenotypes of these isolates were likely due to loss of function of Nrg1.

Given the prevalence of mutations in *NRG1* in the filamentous *C*. *albicans* clinical isolates, we asked whether *NRG1* might also be central to the filamentous phenotype that we observed in three *C*. *parapsilosis* isolates. Indeed, we observed that these isolates each harbored a homozygous mutation in a highly conserved region of the *CpNRG1* gene (R270K), suggesting that perturbation of Nrg1 function is a conserved mechanism for pathogenic yeast that allows filamentation in the absence of inducing cues in clinical isolates from cystic fibrosis patients (S12 Fig).

### Filamentous *C*. *albicans* isolates are resistant to the filamentation-repressing effects of bacterial opportunistic pathogens

**Fig 5:**
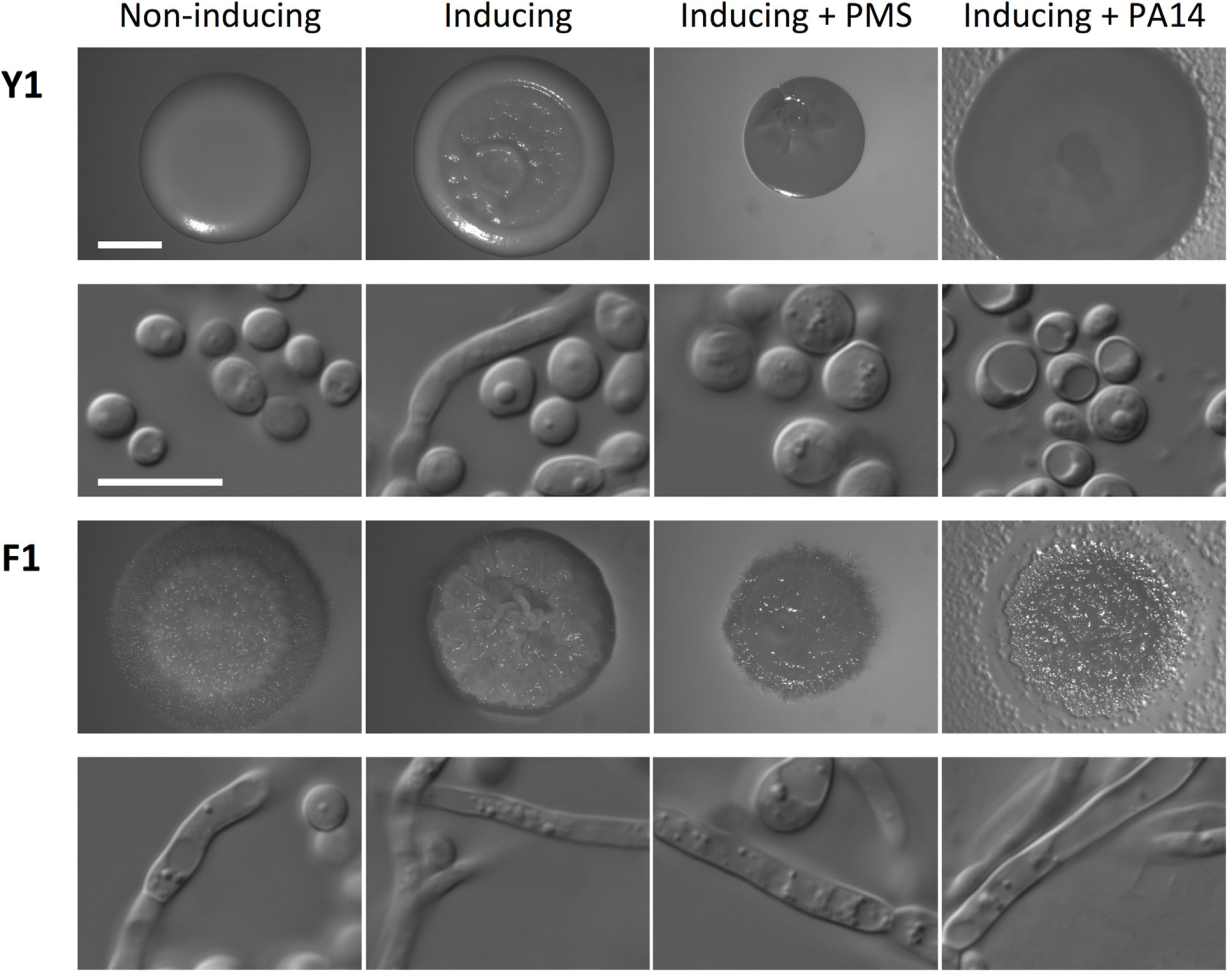
Phenazine methosulfate (PMS) or *P*. *aeruginosa* inhibits filamentation of *C*. *albicans* with standard growth morphology (Y1), but not of filamentous isolate F1. The non-inducing condition is synthetic defined (SD) medium at 30°C and the inducing condition is SD + 5 mM N-acetylglucosamine at 37°C for 48 h. Treatments included 5 μM PMS or 100 μl of PA14 *P*. *aeruginosa* overnight culture, as indicated. Images of colonies are provided in the first and third rows, and DIC microscopy images of cells from the colony are in the second and fourth rows. Scale bar on spot image represents 2 mm, and scale bar on DIC image represents 10 μm.

*P*. *aeruginosa* and *B*. *cenocapacia* can repress filamentation and metabolism of *C*. *albicans* in co-culture conditions via secretion of quorum sensing molecules [20,21]. Given that four out of five patients that produced filamentous *C*. *albicans* isolates (CF028, CF033, CF066, and CF198) were heavily colonized with *P*. *aeruginosa* and patient CF170 was colonized with *Burkholderia multivorans*, which is closely related to *B*. *cenocepacia* [40], we tested whether the filamentous *C*. *albicans* isolate F1 could still form filaments under the repressing conditions of co-culture with *P*. *aeruginosa* or *B*. *multivorans*, or in the presence of a structural analog of pyocyanin, phenazine methosulfate (PMS). *C*. *albicans* isolate Y1 showed the expected smooth colony morphology under standard conditions that promote yeast growth, and the characteristic wrinkled colony morphology under filament-inducing condition (Fig 5). Microscopy images of cells collected from the colonies confirmed that smooth colonies were composed of cells with yeast morphology, and wrinkled colonies were composed of cells with filamentous morphology (Fig 5). In contrast, the F1 filamentous *C*. *albicans* isolate from CF170 showed wrinkled colony morphology under both standard and filament-inducing conditions (Fig 5). Under filament-inducing conditions, the addition of 5 μM PMS or co-culture with *P*. *aeruginosa* reference strain PA14 or *B*. *multivorans* reference strain ATCC17616 inhibited filamentation of Y1, but not of F1 (Fig 5 and S13 Fig). This resistance to filamentation-repressing effects of PMS and *P*. *aeruginosa* was not specific to F1, but was a general feature of all filamentous *C*. *albicans* isolates with mutation in *NRG1* (S14 Fig) and an independently generated *nrg1Δ*/*nrg1Δ* deletion mutant in a different strain background (S15 Fig). Thus, filamentous *C*. *albicans* isolates are resistant to the filamentation-repressing effects of bacterial opportunistic pathogens.

## Discussion

Here, we provide a portrait of species and population level dynamics in the lung mycobiome of cystic fibrosis patients, and illuminate the power of complementary high-throughput sequencing coupled with phenotypic and genotypic analyses. Utilizing high-throughput sequencing of the ribosomal RNA internal transcribed spacer ITS1 amplified from longitudinal sputum samples from 28 cystic fibrosis patients (Fig 1), we were able to identify fungi that were not isolated through culture-based methods, likely due to their specific growth requirements as with lipophilic *Malassezia* species [31] and slow growing *E*. *dermatitidis* [32]. Our culture-based analysis of 1,603 fungal isolates from the same sputum samples revealed extensive inter- and intra-species phenotypic diversity in growth rate, drug resistance, and cellular morphology (Figs 2–4), with profound implications for inter-kingdom microbial interactions and pathogen adaptation to the host (Fig 5). The strong correlation between our molecular and culture-based methods to define fungal communities (Fig 1) provides strong validation of our approach to study species and population level dynamics in the host.

Although studies of the lung mycobiome are in their infancy, there is a growing appreciation that fungi can modulate clinical outcome in the context of chronic respiratory diseases such as cystic fibrosis [1]. We found that different patients harbor distinct fungal communities, although the dominant trend was stable colonization with *Candida* and *Aspergillus* species (Fig 1, S6 and S7 Figs); this is consistent with previous analyses of the cystic fibrosis mycobiome [6,7,13], and distinct from findings with the lung mycobiome of healthy individuals [12]. Neither fungal diversity nor community structure in our patient population was correlated with patient characteristics such as BMI, FEV_1_% predicted values, clinical status, lung disease stage, or antifungal treatments (S2–S7. Figs), consistent with a prior study of the bacterial and fungal microbiome of adult cystic fibrosis patients [13]. However, we did detect a weak yet significant negative correlation between relative abundance of *C*. *albicans* and patient FEV_1_% predicted values (S5 Fig), consistent with a previous association between chronic *C*. *albicans* colonization and FEV_1_ decline in some cystic fibrosis patients [11], reinforcing the relevance of *C*. *albicans* colonization in context of cystic fibrosis. Our finding that the lung mycobiome of adult cystic fibrosis patients is relatively stable over longitudinal sampling periods with little perturbation in response to changes in patient physiology or antibiotic therapies resonates with the emerging theme that bacterial diversity remains relatively stable in cystic fibrosis patients over the course of clinical exacerbation and treatment [13,41]. It is well established that bacterial communities of cystic fibrosis patient lungs differ significantly between adolescents and adults [3], with diversification events prevalent in younger patients and community specialization accompanying the deterioration of pulmonary function with age [42,43]. Thus, analysis of fungal microbiomes in age-stratified cystic fibrosis patients may reveal more dynamic communities and changes associated with disease progression.

Despite relatively stable fungal communities based on taxonomic identities, we identified considerable inter- and intra-species phenotypic variation in traits important for host adaptation. Variation in antifungal drug resistance largely tracked with species identity, and there was no evidence for the evolution of antifungal drug resistance in response to drug treatments (Fig 2, S8 Fig). This stands in contrast to the rapid emergence of drug resistance in fungal populations in the host that has been observed with *Candida* species from patients with AIDS or Crohn’s disease [15,16], and with the high frequency of azole-resistant *A*. *fumigatus* recovered from cystic fibrosis patients treated with azoles [44,45]. This may reflect differences in antifungal treatment regimens, pathogen proliferations rates, or pathogen population sizes in these patients, which can influence the selection pressure for resistance and the probability of accumulating resistance mutations. The most striking phenotype that we identified was the uncoupling of morphogenesis in *Candida* species from regulation by standard inducing cues. The capacity to transition between yeast and filamentous growth is a key virulence trait for *C*. *albicans*, with most mutants that are unable to transition being attenuated in virulence. The current paradigm is that filaments are responsible for tissue invasion and escape from immune cells, while yeasts are critical for dissemination [46] Filaments are further implicated in virulence as they express virulence factors such as adhesins and proteases [47,48]. Our discovery of 25 *C*. *albicans* isolates that filament in the absence of inducing cues (Fig 2A), suggests that there may be a fitness advantage to enabling altered regulation of morphogenesis. That this trait emerged independently in *C*. *albicans* recovered from multiple patients and in *C*. *parapsilosis* (S12 Fig) but has not been reported in other patient populations, underscores that this may be a prevalent adaptation to the cystic fibrosis lung environment. Inter-kingdom interactions in this environment may provide the selective pressure driving this adaptive change, as dominant bacteria such as *P*. *aeruginosa* and *Burkholderia* species repress *C*. *albicans* filamentation [18–21], and the filamentous isolates we recovered are resistant to the repressive effects on morphogenesis (Fig 5, S13 and S14 Figs). The intra-species phenotypic diversity in fungal morphology complements the extensive diversity observed in bacterial populations infecting in the cystic fibrosis lung [49–52], and suggests extensive genetic variation in microbial populations that can enable adaptive evolution in the host.

Adaptation of microbial pathogens in response to host selective pressures over the course of chronic infections manifests in genetic signatures in pathogen populations. This is best appreciated in the context of bacterial pathogens, where adaptation of *P*. *aeruginosa* and *Burkholderia* species during chronic pulmonary infections has been accompanied by the acquisition of adaptive mutations [49–54]. It is clear that many mutations remain polymorphic in the bacterial populations, suggesting limited clonal selective sweeps. Our analysis of the genetic basis of the filamentation phenotype of *Candida* isolates suggests a similar trend in fungal populations. Twenty four of the 25 filamentous *C*. *albicans* isolates and all three filamentous *C*. *parapsilosis* isolates contained homozygous mutations in the transcriptional repressor of filamentation, *NRG1* (Figs 3 and 4, S12 Fig). The `filamentous isolates recovered from a single patient most often all shared the same *nrg1* allele, although there was one patient that harbored *C*. *albicans* isolates with distinct homozygous *nrg1* mutations (Fig 4). This suggests that the filamentation phenotype emerged independently within individual patients, and that the loss of function of *NRG1* is a common mechanism of adaptation to the cystic fibrosis lung environment. Despite the potential fitness advantage of retaining the capacity to filament in the presence of bacterial pathogens, the frequency of filamentous isolates remained low. This may reflect a bias in culturing the filamentous isolates if they remain embedded in tissues thereby minimizing recovery from sputum samples. Alternatively, it could reflect that the magnitude of fitness benefit is small or that fungal proliferation is limited such that the mutants would not sweep to fixation. Yet another possibility is that loss of function of *NRG1* may confer niche-specific fitness advantages, consistent with the observed heterogeneity in bacterial populations that has been attributed to spatial and temporal heterogeneity in the cystic fibrosis lung environment [55–58]. Experimental evolution studies clearly demonstrate that structured physical, nutritional, and cooperative niches can select for the evolution of distinct populations [59–61]. Our finding that the majority of filamentous isolates harbored mutations in *NRG1* rather than loss-of-function mutations in other transcriptional repressors such as *TUP1* or *RFG1* [37,62,63], suggests that there may be distinct fitness consequences of mutation of these different transcriptional repressors of filamentation. The complexity of microbial evolution in the host is likely to be exquisitely contingent upon interactions among constituents of the microbiome, and reflects the stunning diversity of adaptive strategies in biological systems.

## Materials and Methods

### Clinical specimen collection

Protocols for the collection and use of cystic fibrosis patient sputum were approved by the Research Ethics Boards of St. Michael's Hospital and the University Health Network. Informed consent was obtained from each study subject and all sputum specimens were produced voluntarily. Experiments involving patient specimens were conducted in accordance with the *Tri-Council Policy Statement*: *Ethical Conduct for Research Involving Humans*, of the Canadian Institutes of Health Research (CIHR), the Natural Sciences and Engineering Research Council of Canada (NSERC) and the Social Sciences and Humanities Research Council of Canada (SSHRC).

### ITS1 sequencing

The ITS1 region of the 18S-ITS1-5.8S-ITS2-28S rRNA complex was amplified from the DNA extracted from sputum using the ITS1F and ITS1R PCR primer set [13]. PCR reactions were performed in triplicate and pooled. The samples were cycled at 95°C for 3 minutes, 30 cycles of 95°C for 15 seconds, 56°C for 15 seconds, 72°C for 15 seconds, followed by a final extension at 72°C for 5 minutes. The amplicons were cleaned using AMPure XP magnetic beads (Beckman Coulter, Inc.) and prepared for sequencing using the Nextera XT DNA library preparation kit following the manufacturer instructions (Illumina, Inc.). Samples were pooled and size selected on a 1% TAE (w/v) agarose gel. The library was sequenced on the Illumina Miseq using a 150x2 PE sequencing kit.

Following the UPARSE pipeline, the sequencing reads were assembled, quality filtered and dereplicated [64]. Operational Taxonomic Units (OTUs) were then clustered into groups of ≥97% sequence identity and chimeras were removed. Taxonomic identity was assigned to the OTUs using BLASTn and the FHiTINGS v.1-2 reference database [65,66]. The BLASTn results were input into a modified version of the FHiTINGS program to identify the taxonomy based on BLAST hit frequency, e-value scores and common taxonomic ancestors. The taxonomies and the OTU abundances were converted into an OTU table using the biom-format V1.3.1 software [67]. OTUs with <0.005% relative abundances were removed [68]. For downstream analyses QIIME (Version 1.9.0) was used [67]. Raw relative abundances were used to generate taxonomic abundance plots then the reads were rarefied at 1,000 reads for calculating Simpson’s diversity index and Bray-Curtis dissimilarity index. Principal coordinate plots were generated for sputum samples from patients with more than one sampling time point. Downstream statistical analyses were performed on R (Version 3.1.0).

### Recovery of fungal isolates

Sputa were collected by expectoration and transported to the laboratory on ice. Sputa were solubilized by homogenization with Sputolysin (EMD Millipore) and separate aliquots were used for mycobiome and fungal analysis. To culture fungus, duplicate aliquots of each sample were plated directly onto Sabouraud Dextrose Agar (SDA) (Becton Dickinson) supplemented with 50 μg/mL ampicillin (Sigma) and 50 μg/mL kanamycin (Sigma) without serial dilution. All cultures were incubated at 37°C for 48 h. Following incubation, colonies were presumptively identified as fungus by visual inspection and up to 50 colonies were selected from each sample for further analysis by morphology. If there were less than 50 fungal colonies from plating a sputum sample, all colonies were included in the sampling. If there were more than 50 fungal colonies from plating a sputum sample, an effort was made to maintain the relative abundance of each colony morphology in the sampling. Fungal isolates were then cryopreserved at -80°C in 25% glycerol (v/v) after a single subculture in LB broth (Wisent Inc.).

### Screening mold isolates

Mold isolates were grown on individual potato dextrose agar (PDA) plates containing 100 μg/ml ampicillin (BioShop) and 50 μg/ml gentamicin (BioShop) and incubated at 37°C for up to 72 hours until robust sporulation was observed. Spores were harvested by gently washing the plates with sterile water and passing through Miracloth filter. After pelleting the spores and re-suspending in sterile water, optical density at 600 nm (OD_600_) was measured using a spectrophotometer. Spore suspensions were diluted to the final OD_600_ of 0.01 in 100 μl of RPMI1640 or 100 μl of RPMI1640 containing 0.5 μg/ml itraconazole (Sigma) or 1 μg/ml amphotericin B (Sigma) in clear 96-well plate, covered with clear, adhesive seal (Thermo Scientific). Growth was measured by OD_595_ inside GENios microplate reader (TECAN) every 15 min for 48 hours at 37°C.

Species profiles were generated by the combination of morphological characterization and ITS2 sequencing of representative isolates. The ITS2 locus was PCR amplified directly from spore suspensions with primers oLC2459 and oLC2460. Reaction mixtures contained 1x PCR buffer, 0.25 mM deoxynucleotide triphosphates (dNTPs), 0.5 mM primers, 1 unit of Taq polymerase, 5 μl of spore suspension, and sterile water up to 20 μl. Cycling conditions were 98°C 2 min; 98°C 20 s, 55°C 20 s, and 72°C 20 s for 30 cycles; and 72°C 2 min. PCR products were visually confirmed by gel electrophoresis and purified using PCR cleanup kit (Sigma). Purified products were sent for Sanger sequencing with 100 ng of product and 7.14 μM of oLC2459 at TCAG sequencing facility (Toronto, ON). Using BLAST [66], ITS2 sequences were queried against NCBI nucleotide database and species identity was assigned based on the highest bit score sequence. Once species identity was assigned, all isolates from the same patient with the same growth morphologies were classified as the same species.

### Screening yeast isolates

Yeast isolates were grown on individual yeast extract peptone dextrose (YPD: 1% yeast extract, 2% bactopeptone, 2% glucose) plates containing 100 μg/ml ampicillin and 50 μg/ml kanamycin (BioShop) and incubated for 24 hours at 30°C. Yeast isolates were subsequently sub-cultured in 200 μl of YPD in clear 96-well plate overnight at 30°C. Overnight cultures were diluted 20,000 fold into 100 μl of YPD in clear 96-well plates and covered with clear, adhesive seals. Growth was measured by OD_595_ every 15 min using a GENios microplate reader with rotation at 800 rpm for 36 hours at 30°C. The same overnight cultures were diluted 20,000 fold into 200 μl of YPD and 200 μl YPD containing 128 μg/ml fluconazole (Sequoia Research Products) in clear 96-well plates. Final growth was measured by OD_600_ after 48 h at 30°C using a spectrophotometer (Molecular Devices).

Species profiles were generated using both CHROMagar Candida (BD, 254093) and ITS2 sequencing of representative isolates. All isolates were spotted on CHROMagar Candida for identification of the most prevalent *Candida* species (*C*. *albicans*, *C*. *tropicalis*, *C*. *glabrata*, *C*. *parapsilosis*) [28]. The ITS2 locus was PCR amplified from isolates representative of each species as with the mold isolate screening method above, but colony suspensions were used instead of spore suspensions.

### Culture conditions for *Candida* filamentation

All *Candida* isolates were typically grown in YPD medium overnight at 30°C in shaking conditions, unless stated otherwise. Solid media plates were supplemented with 1% agar.

The effects of PMS (Sigma) on *C*. *albicans* filamentation were tested as described in Morales *et al*., 2013 [20]. The non-inducing condition is YNB (BioShop) + 10 mM Glucose (BioShop) + 0.2% amino acids (BioShop) at 30°C and the inducing condition is YNB + 10 mM Glucose + 0.2% amino acids 5 mM N-acetylglucosamine (BioBasic) at 37°C for 48 hours.

Cells were visualized by imaging 5 μl of cultures at specific time points or suspended cells from colonies using DIC microscopy (Zeiss Axio Imager.MI, Carl Zeiss). Colonies were visualized using an M2 Discovery Stereomicroscope (Carl, Zeiss).

### Sputum DNA extraction

Sputum DNA was extracted using MasterPure Yeast DNA Purification Kit (Epicentre) following the manufacturer’s instructions with a minor modification. To physically disrupt fungal cells, 50 μl of sputum was mixed with 300 μl Yeast Lysis Solution and 200 μl acid-washed glass beads and homogenized in a bead beater for 3 min. Mock preparations were performed in parallel with sputum samples in order to ensure that the reagents were free of contaminating fungal DNA.

DNA was quantified using Quant-iT PicoGreen dsDNA Assay Kit (Life Technologies) following the manufacturer’s protocol. Fluorescence was measured with excitation wavelength at 480 nm and emission wavelength at 520 nm on the spectrophotometer (Molecular Devices). DNA concentration was calculated based on the standard curve.

### Whole genome sequencing of clinical isolates and SNV confirmation

Cell pellets of clinical isolates were prepared by centrifuging 20 ml of overnight culture at 3,000 rpm for 5 min and flash frozing using dry ice and ethanol. Sequencing libraries were prepared using the Nextera XT Kit (Illumina) according to the manufacturer’s instructions. Libraries were sequenced on the Illumina MiSeq platform using paired reads (150 bp). The sequence reads were de-multiplexed and trimmed to remove bases with Phred scores < Q30. Reads were aligned to SC5314 reference genome using Bowtie2 (Version 2.0.7) [69], and the alignment was visualized using Savant Genome Browser [70]. MuTect (Version 1.1.4) [36] was used to identify unique mutations in filamentous *C*. *albicans* isolates. MuTect provides accurate variant detection in diploid genomes based on a model that takes into account the matched normal (not mutated) DNA, as well as sequencing errors and allele fractions. The sequence data is publicly available on the NCBI Sequence Read Archive with accession number SRX1084067.

Mutations in *NRG1* were further confirmed by PCR amplifying *NRG1* from genomic DNA (gDNA) of clinical isolates using primers oLC3080 and oLC3282. Reaction mixtures contained 1x PCR buffer, 0.25 mM dNTPs, 0.5 mM primers, 1 unit of Taq polymerase, 100 ng of gDNA, and sterile water up to 20 μl. Cycling conditions were 98°C 2 min; 98°C 20 s, 55°C 20 s, 72°C 20 s for 30 cycles; and 72°C 2 min. PCR products were visually confirmed by gel electrophoresis and purified using a PCR cleanup kit (Sigma). Purified products were sent for Sanger sequencing with 100 ng of product and 7.14 μM of oLC3080 and oLC3282 at TCAG sequencing facility.

### Plasmid and strain construction

*E*. *coli* DH5α competent cells were used for plasmid construction. Strains of bacteria and *C*. *albicans* that were used engineered in this study are listed in S4 Table and oligonucleotides in S5 Table.

To construct a cassette for *NRG1* allele replacements, part of the *NRG1* open reading frame spanning the polymorphism identified and the *NRG1* downstream region were amplified from *C*. *albicans* strain Y1 and F1 gDNA, using oLC3093/oLC3155 (768 bp) and oLC3094/oLC3095 (544 bp). The amplified region of the open reading frame was cloned into pLC49 [71] at KpnI and ApaI, which is upstream of the nourseothricin (NAT) resistance marker and FLP recombinase. The *NRG1* downstream region was then cloned in at SacI/SacII, which is downstream of the NAT resistance marker and FLP recombinase. The presence of the *NRG1* open reading frame and the *NRG1* downstream region was tested by PCR with oLC275/M13R (970 bp) and oLC274/M13F (736 bp), respectively. The final constructs were sequence verified using the same set of primers used to generate the amplicons. The construct to integrate the Y1 *NRG1* allele is pLC796 and the construct to integrate the F1 *NRG1* allele is pLC798. Bacteria harbouring the plasmid are propagated with ampicillin (100 μg/ml) and nourseothricin (250 μg/ml) (NAT, Werner BioAgents). Using KpnI and SacI, the cassette was liberated for transformation.

For *C*. *albicans* transformations, 1 ml of an overnight YPD culture with an OD_600_ of between 4–8 was used. Cells were pelleted and resuspended with the following transformation mixture: 1.5 μg of digested DNA, 40% polyethylene glycol (PEG), 1X Tris-EDTA, 100 mM lithium acetate, pH 7.4, 10 mg/ml of salmon sperm DNA, and 20 mM dithiothreitol (DTT). The mixture was incubated at 30°C for 1 hour and 42°C for 45 min. Cells were washed with 1 ml YPD and re-suspended in 10 ml YPD and allowed to recover at 30°C shaker for 4 hours. Transformants were selected for on YPD plates containing NAT and incubated at 30°C for 48 hours. The FLP recombinase was induced to excise the NAT cassette by growth in yeast nitrogen base bovine serum albumin (YNB-BSA) medium at 30°C with shaking for 48 hours. Approximately 100 cells were plated on a YPD plate and incubated at 30°C for 48 h, then replica plated onto YPD + NAT and incubated at 30°C overnight to identify NAT-sensitive colonies.

The Y1 *NRG1/nrg1Δ* mutant was generated by PCR amplification of the NAT-FLP cassette from pLC49 [71] with primers oLC3112/oLC3113 (4366 bp), which contain homology to precisely replace the *NRG1* open reading frame. Upstream and downstream integration of the cassette were tested by PCR using oLC275/oLC3080 (635 bp) and oLC274/3155 (357 bp), respectively. The NAT marker was excised and the genotype verified by PCR using oLC3080/oLC3155 (833 bp deletion allele and 1510 bp native allele).

## Supporting Information

S1 Fig Cystic fibrosis lung mycobiome at the genus level. Relative abundance of species in 89 sputum samples from 28 patients at genus level, identified by culture (top) and ITS1 sequencing (bottom). *: Pearson correlation > 0.5.
(TIFF)

S2 Fig Simpson’s diversity index does not correlate with patient clinical characteristics. (A) Clinical state in BETR categories as described in Zhao et al., 2012 [72]. B: baseline–well or mild increase in pulmonary symptoms; not a doctor defined pulmonary exacerbation; not hospitalized for increase in pulmonary symptoms; not on episodic antibiotics for > 30 days; may or may not be on maintenance antibiotics. E: exacerbation–doctor defined pulmonary exacerbation or increased pulmonary symptoms fitting definition of exacerbation; before start of episodic IV or oral antibiotics; not on episodic antibiotics for >30 days; may or may not be on maintenance antibiotics. T: treatment–on IV or oral episodic antibiotics for treatment of doctor defined exacerbation or increased pulmonary symptoms fitting definition of exacerbation. R: recovering–off episodic antibiotics < 30 days; may or may not be on maintenance antibiotics; may or may not be back to baseline clinical state. O: other–patient is on antibiotic therapy for something other than a pulmonary exacerbation. B-F: baseline, but drop in lung function–clinic notes indicate drop in lung function, but no antibiotic prescription was given that day for treatment or other complications. (B) Lung disease state based on FEV% prediction. Early: >70; Intermediate: between 70 to 40; Advanced: <40. If FEV% prediction was not available, lung disease state was not calculated. (C) Clinical microbiology identification of *Pseudomonas*. (D) Clinical microbiology identification of mucoid *Pseudomonas*. (E) Triazole prescription. N: on azole prescription at the time of sampling; Y: on azole prescription at the time of sampling. Kruskal-Wallis one-way ANOVA.
(TIFF)

S3 Fig Shannon diversity index does not correlate with patient clinical characteristics. (A) Clinical state in BETR categories as described in Zhao et al., 2012 [72]. B: baseline–well or mild increase in pulmonary symptoms; not a doctor defined pulmonary exacerbation; not hospitalized for increase in pulmonary symptoms; not on episodic antibiotics for > 30 days; may or may not be on maintenance antibiotics. E: exacerbation–doctor defined pulmonary exacerbation or increased pulmonary symptoms fitting definition of exacerbation; before start of episodic IV or oral antibiotics; not on episodic antibiotics for >30 days; may or may not be on maintenance antibiotics. T: treatment–on IV or oral episodic antibiotics for treatment of doctor defined exacerbation or increased pulmonary symptoms fitting definition of exacerbation. R: recovering–off episodic antibiotics < 30 days; may or may not be on maintenance antibiotics; may or may not be back to baseline clinical state. O: other–patient is on antibiotic therapy for something other than a pulmonary exacerbation. B-F: baseline, but drop in lung function–clinic notes indicate drop in lung function, but no antibiotic prescription was given that day for treatment or other complications. (B) Lung disease state based on FEV% prediction. Early: >70; Intermediate: between 70 to 40; Advanced: <40. If FEV% prediction was not available, lung disease state was not calculated. (C) Clinical microbiology identification of *Pseudomonas*. (D) Clinical microbiology identification of mucoid *Pseudomonas*. (E) Triazole prescription. N: on azole prescription at the time of sampling; Y: on azole prescription at the time of sampling. Kruskal-Wallis one-way ANOVA.
(TIFF)

S4 Fig Simpson’s and Shannon diversity indices do not correlate with patient FEV_1_% predicted values or BMI. FEV_1_% predicted values and BMI compared to (A) Simpson’s or (B) Shannon diversity index. Linear regression.
(TIFF)

S5 Fig *C*. *albicans* relative abundance is negatively correlated with patient FEV_1_% predicted values. Linear regression.
(TIFF)

S6 Fig Principal coordinate visualization of the Bray-Curtis dissimilarity index of patient sputum samples and patient characteristics. (A) Patient ID. (B) Clinical state. B: baseline–well or mild increase in pulmonary symptoms; not a doctor defined pulmonary exacerbation; not hospitalized for increase in pulmonary symptoms; not on episodic antibiotics for > 30 days; may or may not be on maintenance antibiotics; E: exacerbation–doctor defined pulmonary exacerbation or increased pulmonary symptoms fitting definition of exacerbation; before start of episodic IV or oral antibiotics; not on episodic antibiotics for >30 days; may or may not be on maintenance antibiotics; T: treatment–on IV or oral episodic antibiotics for treatment of doctor defined exacerbation or increased pulmonary symptoms fitting definition of exacerbation; R: recovering–off episodic antibiotics < 30 days; may or may not be on maintenance antibiotics; may or may not be back to baseline clinical state; O: other–patient is on antibiotic therapy for something other than a pulmonary exacerbation; B-F: baseline, but drop in lung function–clinic notes indicate drop in lung function, but no antibiotic prescription was given that day for treatment or other complications. (C) Lung disease stage based on FEV % prediction. Early: >70; Intermediate: between 70 to 40; Advanced: <40.
(TIFF)

S7 Fig Principal coordinate visualization of the Bray-Curtis dissimilarity index of patient sputum samples and *Pseudomonas* colonization and triazole prescription. (A) *Pseudomonas* load. (B) Mucoid *Pseudomonas* load. (C) Triazole prescription.
(TIFF)

S8 Fig Azole susceptibility phenotypes of yeast isolates recovered from cystic fibrosis patients who received azole treatment. Each dot represents a yeast isolate from the corresponding cystic fibrosis patient and colours represent species identity. The vertical dispersion of isolates within each phenotypic class is simply for visual clarity, as with the horizontal dispersion of isolates from an individual patient. The black line indicates the duration of azole treatment. (A) Summary of isolates from patient 006. (B) Summary of isolates from patient 025. (C) Summary of isolates from patient 107. The isolates are categorized as: “Fluconazole resistant” if their relative growth with fixed concentration of fluconzaole at 128 μg/ml was greater than 2 times that of the relative growth of reference *C*. *albicans* strain SN95; and “Fluconazole susceptible” if their relative growth was less than 2 times the relative growth of SN95.
(TIFF)

S9 Fig Amphotericin B susceptibility phenotypes of mold isolates from cystic fibrosis patients. Each dot represents a mold isolate from the corresponding cystic fibrosis patient and colours represent species identity. The isolates are categorized as: “Amphotericin B resistant” if their relative growth with fixed concentration of amphotericin B at 1 μg/ml was greater than 20% of the no drug control; and “Amphotericin B susceptible” if their relative growth was less than 20% of the no drug control. The vertical dispersion of isolates within each phenotypic class is simply for visual clarity, as with the horizontal dispersion of isolates from an individual patient.
(TIFF)

S10 Fig Growth characteristics of yeast isolates. Kinetic measurements of OD_600_ and DIC images of the reference strain and representative clinical isolates. Scale bar represents 50 μm.
(TIFF)

S11 Fig Growth characteristics of mold isolates. Kinetic measurements of OD_600_ and DIC images of the reference isolate and representative clinical isolates. Scale bar represents 50 μm.
(TIFF)

S12 Fig Filamentous *C*. *parapsilosis* isolates harbour mutations in *NRG1*. (A) DIC microscopy images of three filamentous *C*. *parapsilosis* isolates. Scale bar represents 20 μm. (B) MUSCLE alignment of the C_2_H_2_ DNA binding region of *NRG1* from closely related yeast species. Arrows indicate the mutations identified from filamentous *C*. *albicans* and *C*. *parapsilosis* isolates.
(TIFF)

S13 Fig *B*. *multivorans* inhibits filamentation of *C*. *albicans* with standard growth morphology (Y1), but not the filamentous isolate F1. The non-inducing condition is synthetic defined (SD) medium at 30°C and the inducing condition is SD + 5 mM N-acetylglucosamine at 37°C for 48 hours. For the *B*. *multivorans* (BM) treatment, 100 μl of ATCC17616 *B*. *multivorans* overnight culture was included. Images of colonies are provided in the first and third rows, and DIC microscopy images of cells from the colony are in second and fourth rows. Scale bar on spot image represents 2 mm, and scale bar on DIC image represents 50 μm.
(TIFF)

S14 Fig Filamentous *C*. *albicans* isolates from multiple patients are resistant to the repressing effects of phenazine methosulfate (PMS) or *P*. *aeruginosa*. The non-inducing condition is synthetic defined (SD) medium at 30°C and the inducing condition is SD + 5 mM N-acetylglucosamine at 37°C for 48 hours. For the PMS treatment, 5 μM PMS was included, and for the PA14 treatment, 100 μl of PA14 *P*. *aeruginosa* overnight culture was included. Images of colonies are provided in the first and third rows, and DIC microscopy images of cells from the colony are in second and fourth rows. Scale bar on spot image represents 2 mm, and scale bar on DIC image represents 10 μm. Representative filamentous isolates from three different patients are shown. (A) CF028 isolate NP1B6 (Q118* in *NRG1*). (B) CF033 isolate NP1E3 (Y138* in *NRG1*). (C) CF066 isolate NP1E12 (C233Y in *NRG1*).
(TIFF)

S15 Fig Targeted *nrg1Δ*/*nrg1Δ* deletion mutants are resistant to the filamentation-repressive effects of phenazine methosulfate (PMS) or *P*. *aeruginosa*. The non-inducing condition is synthetic defined (SD) medium at 30°C and the inducing condition is SD + 5 mM N-acetylglucosamine at 37°C for 48 hours. For the PMS treatment, 5 μM PMS was included, and for the PA14 treatment, 100 μl of PA14 *P*. *aeruginosa* overnight culture was included. Images of colonies are provided in the first and third rows, and DIC microscopy images of cells from the colony are in the second and fourth rows. Scale bar on spot image represents 2 mm, and scale bar on DIC image represents 10 μm. (A) Wild-type parental strain. (B) *nrg1Δ/nrg1Δ* deletion mutant.
(TIFF)

S1 Table Patient clinical profiles and corresponding sputum samples. (XLSX)

S2 Table Mutations identified by whole genome sequencing of the F1 filamentous *C*. *albicans* isolate. (XLSX)

S3 Table Mutations identified by whole genome sequencing of the filamentous *C*. *albicans* isolate without an *NRG1* mutation. (XLSX)

S4 Table Bacterial and fungal strains used in this study. (XLSX)

S5 Table Oligonucleotides used in this study. (XLSX)

S1 File Summary of phenotypic analyses of all fungal isolates. (XLSX)
