# Supplementary figures and images for: Global Analysis of the Fungal Microbiome in Cystic Fibrosis Patients Reveals Loss of Function of the Transcriptional Repressor Nrg1 as a Mechanism of Pathogen Adaptation
Source: PLoS Pathog. 2015 Nov 20;11(11):e1005308. doi: 10.1371/journal.ppat.1005308 (PMC4654494; doi:10.1371/journal.ppat.1005308)

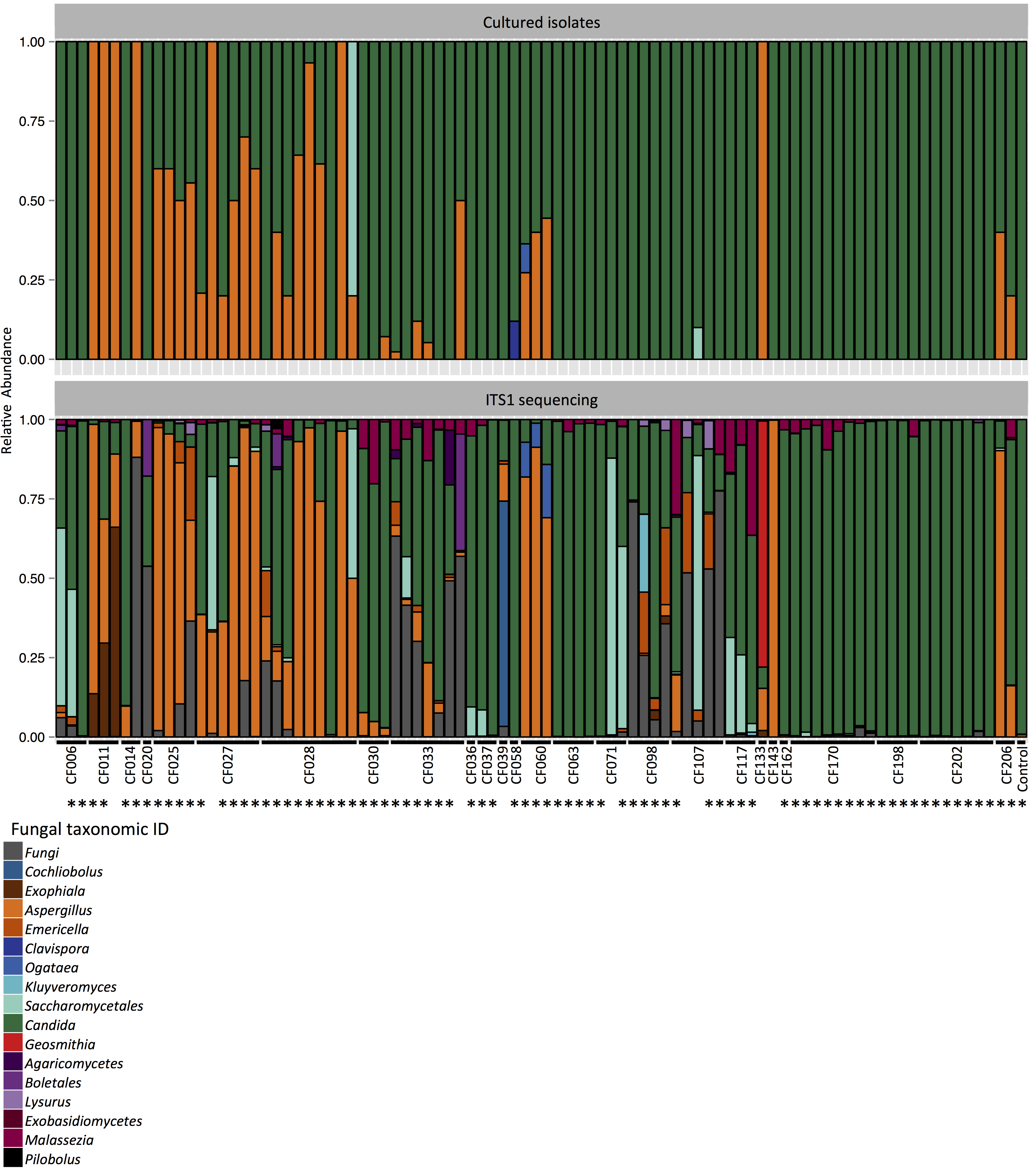

Supplement: S1 Fig — Relative abundance of species in 89 sputum samples from 28 patients at genus level, identified by culture (top) and ITS1 sequencing (bottom). *: Pearson correlation > 0.5. (TIFF) [file ppat.1005308.s001.tiff]

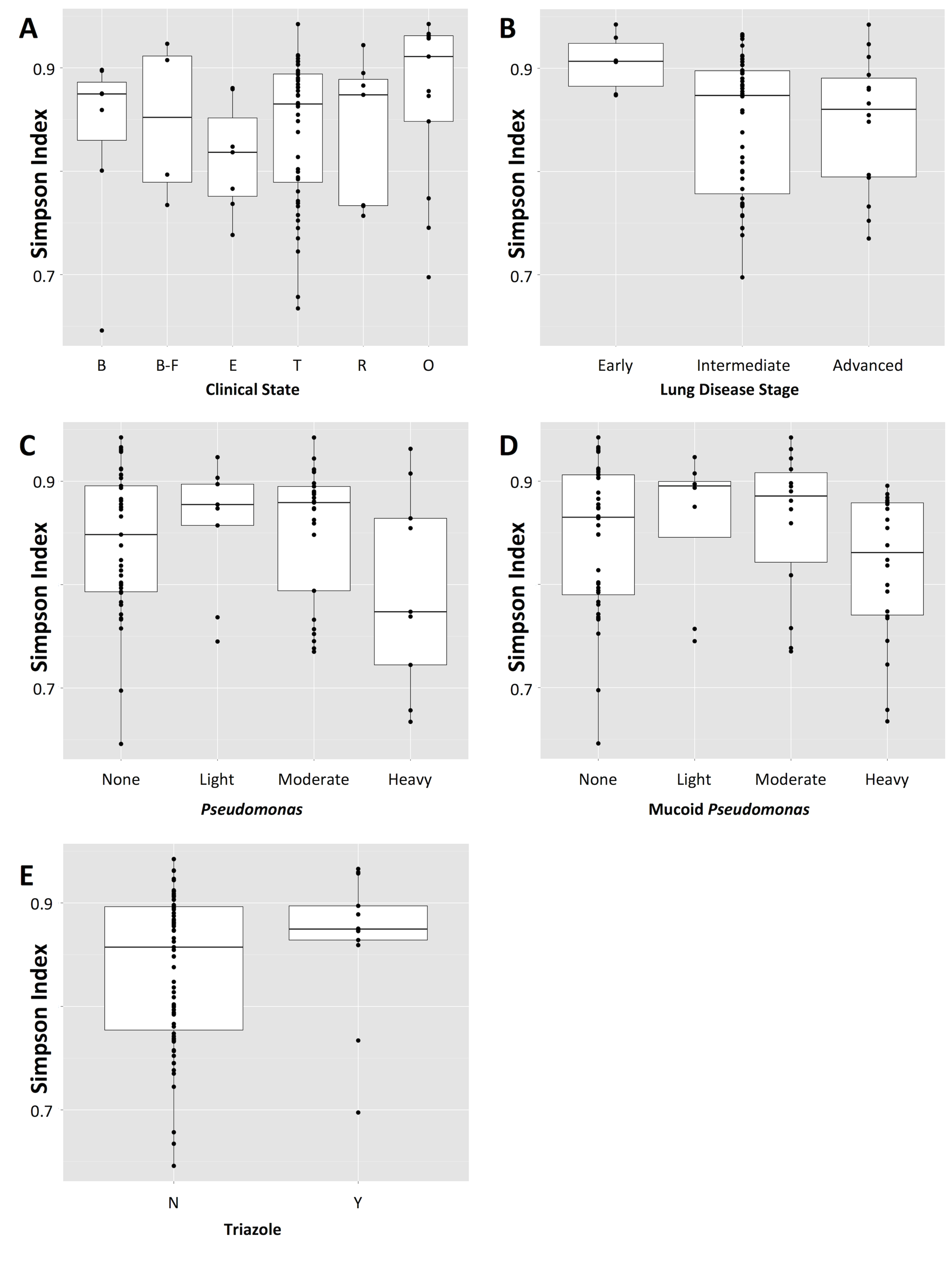

Supplement: S2 Fig — (A) Clinical state in BETR categories as described in Zhao et al., 2012 [72]. B: baseline–well or mild increase in pulmonary symptoms; not a doctor defined pulmonary exacerbation; not hospitalized for increase in pulmonary symptoms; not on episodic antibiotics for > 30 days; may or may not be on maintenance antibiotics. E: exacerbation–doctor defined pulmonary exacerbation or increased pulmonary symptoms fitting definition of exacerbation; before start of episodic IV or oral antibiotics; not on episodic antibiotics for >30 days; may or may not be on maintenance antibiotics. T: treatment–on IV or oral episodic antibiotics for treatment of doctor defined exacerbation or increased pulmonary symptoms fitting definition of exacerbation. R: recovering–off episodic antibiotics < 30 days; may or may not be on maintenance antibiotics; may or may not be back to baseline clinical state. O: other–patient is on antibiotic therapy for something other than a pulmonary exacerbation. B-F: baseline, but drop in lung function–clinic notes indicate drop in lung function, but no antibiotic prescription was given that day for treatment or other complications. (B) Lung disease state based on FEV% prediction. Early: >70; Intermediate: between 70 to 40; Advanced: <40. If FEV% prediction was not available, lung disease state was not calculated. (C) Clinical microbiology identification of Pseudomonas. (D) Clinical microbiology identification of mucoid Pseudomonas. (E) Triazole prescription. N: on azole prescription at the time of sampling; Y: on azole prescription at the time of sampling. Kruskal-Wallis one-way ANOVA. (TIFF) [file ppat.1005308.s002.tiff]

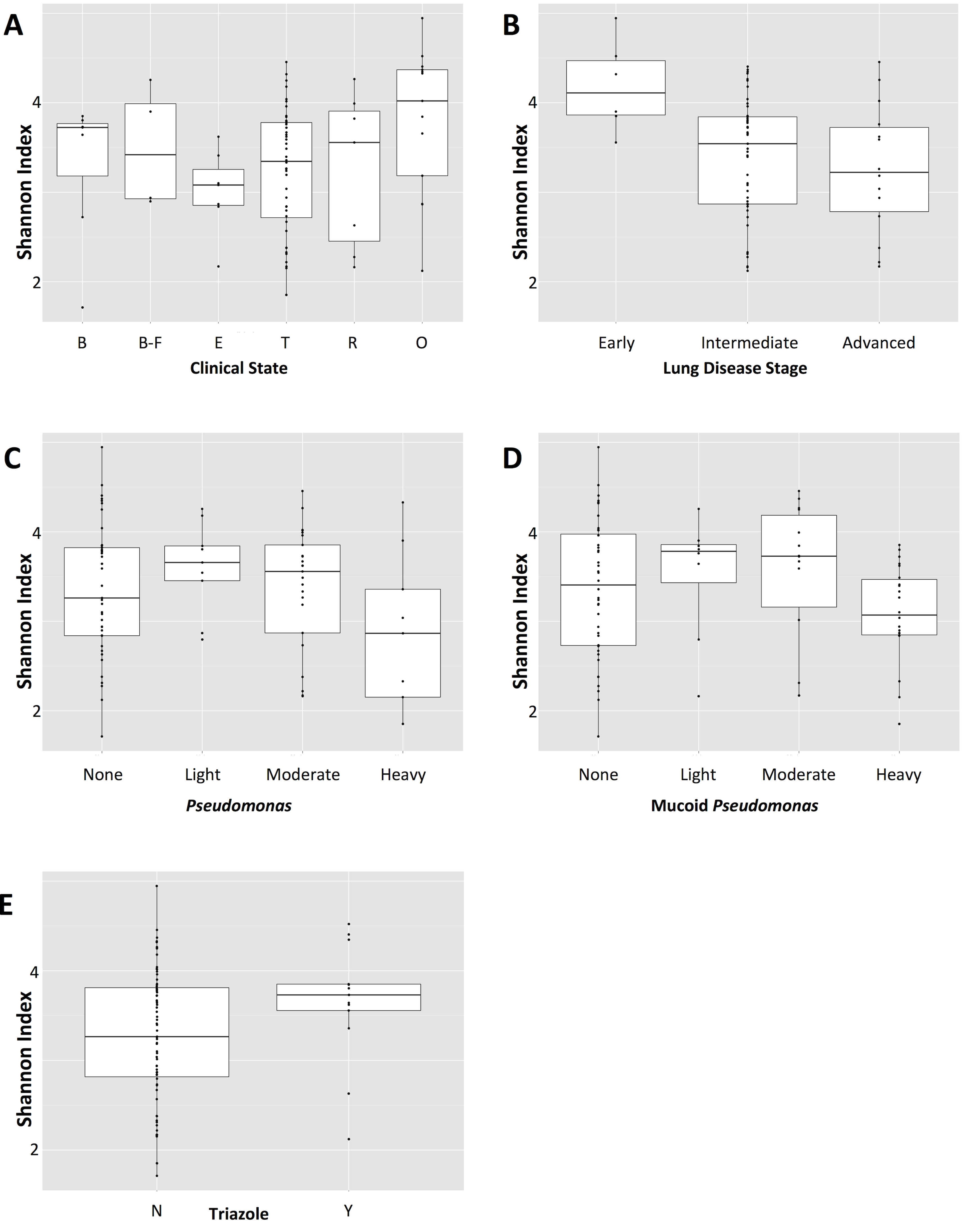

Supplement: S3 Fig — (A) Clinical state in BETR categories as described in Zhao et al., 2012 [72]. B: baseline–well or mild increase in pulmonary symptoms; not a doctor defined pulmonary exacerbation; not hospitalized for increase in pulmonary symptoms; not on episodic antibiotics for > 30 days; may or may not be on maintenance antibiotics. E: exacerbation–doctor defined pulmonary exacerbation or increased pulmonary symptoms fitting definition of exacerbation; before start of episodic IV or oral antibiotics; not on episodic antibiotics for >30 days; may or may not be on maintenance antibiotics. T: treatment–on IV or oral episodic antibiotics for treatment of doctor defined exacerbation or increased pulmonary symptoms fitting definition of exacerbation. R: recovering–off episodic antibiotics < 30 days; may or may not be on maintenance antibiotics; may or may not be back to baseline clinical state. O: other–patient is on antibiotic therapy for something other than a pulmonary exacerbation. B-F: baseline, but drop in lung function–clinic notes indicate drop in lung function, but no antibiotic prescription was given that day for treatment or other complications. (B) Lung disease state based on FEV% prediction. Early: >70; Intermediate: between 70 to 40; Advanced: <40. If FEV% prediction was not available, lung disease state was not calculated. (C) Clinical microbiology identification of Pseudomonas. (D) Clinical microbiology identification of mucoid Pseudomonas. (E) Triazole prescription. N: on azole prescription at the time of sampling; Y: on azole prescription at the time of sampling. Kruskal-Wallis one-way ANOVA. (TIFF) [file ppat.1005308.s003.tiff]

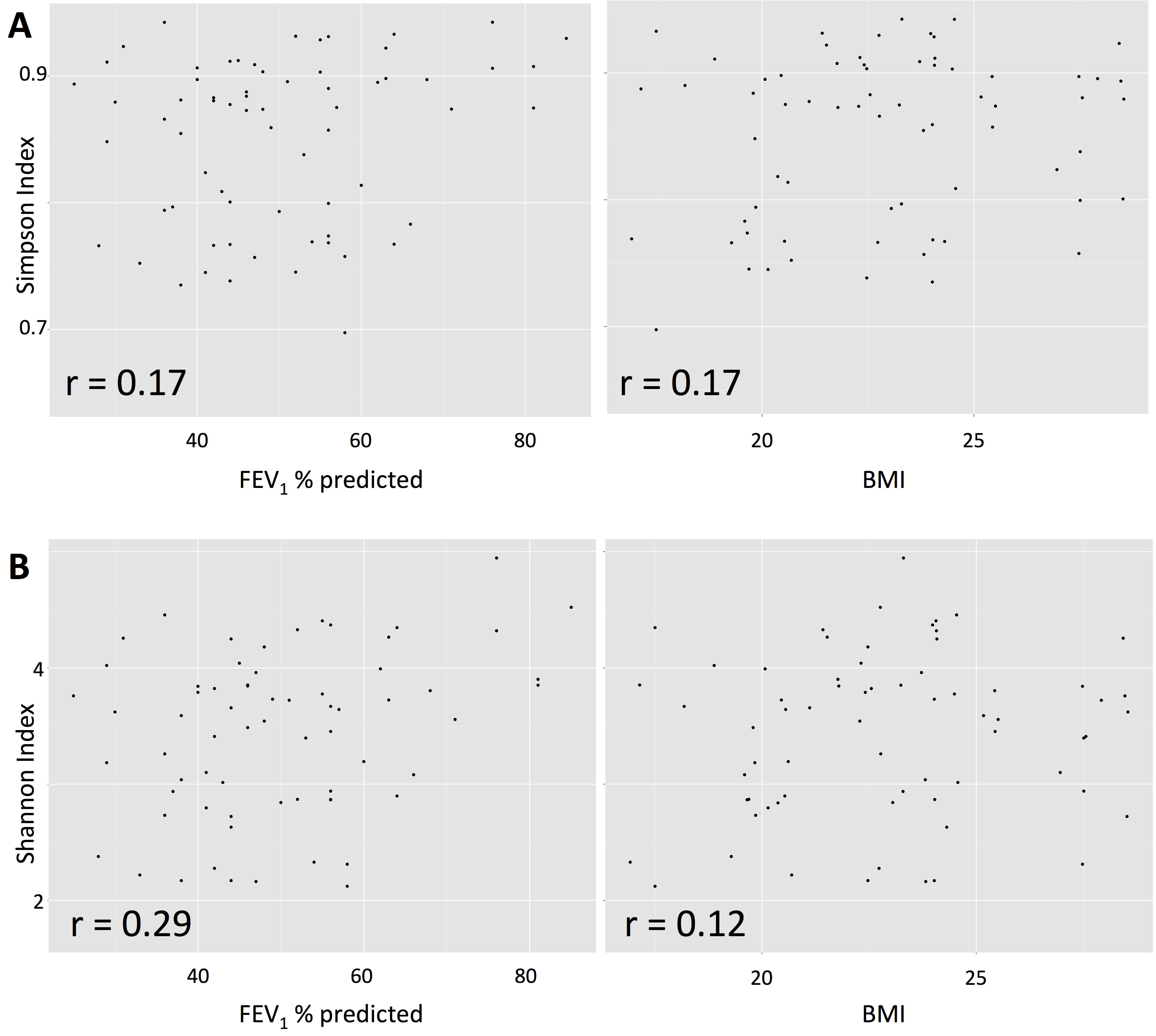

Supplement: S4 Fig — FEV1% predicted values and BMI compared to (A) Simpson’s or (B) Shannon diversity index. Linear regression. (TIFF) [file ppat.1005308.s004.tiff]

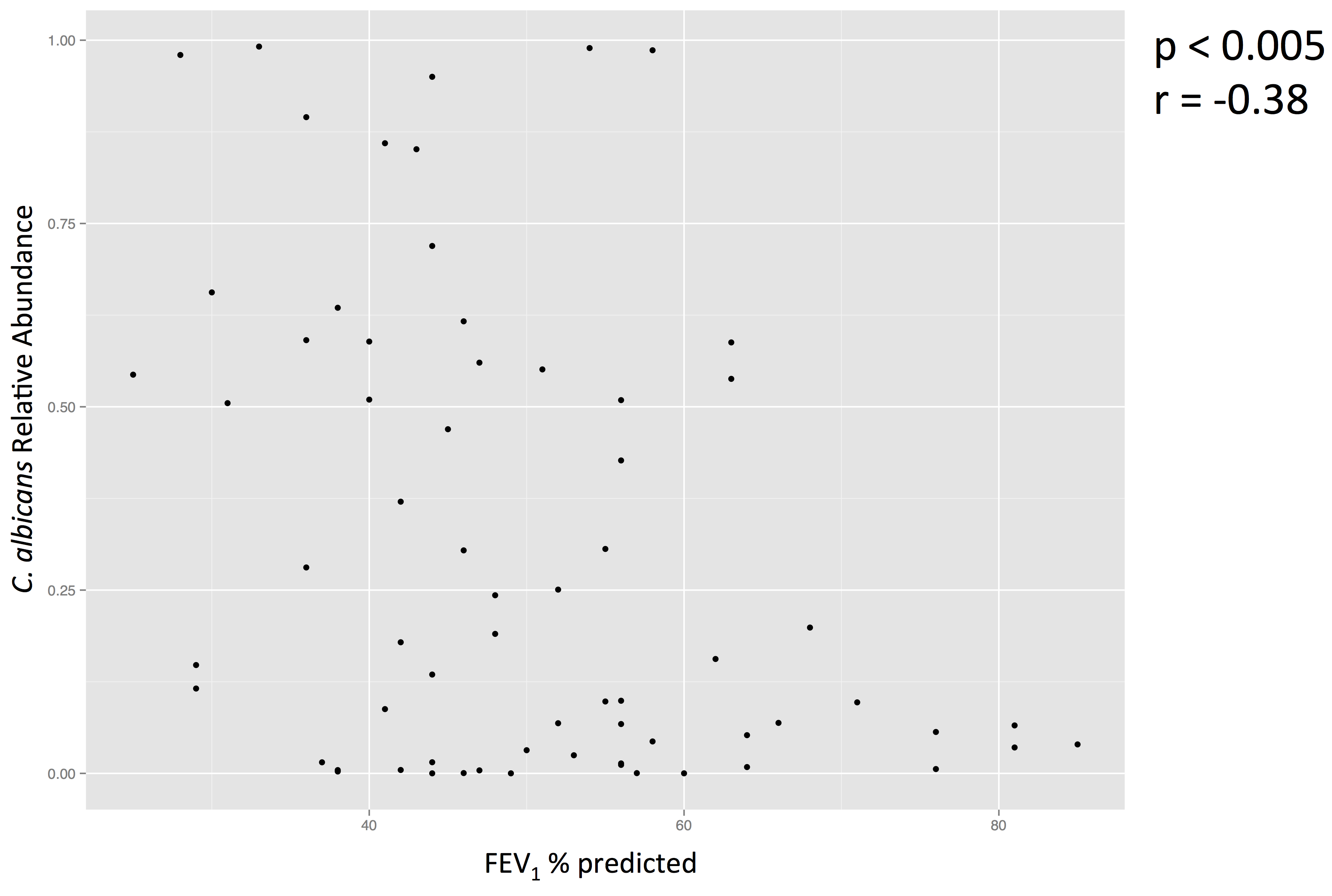

Supplement: S5 Fig — Linear regression. (TIFF) [file ppat.1005308.s005.tiff]

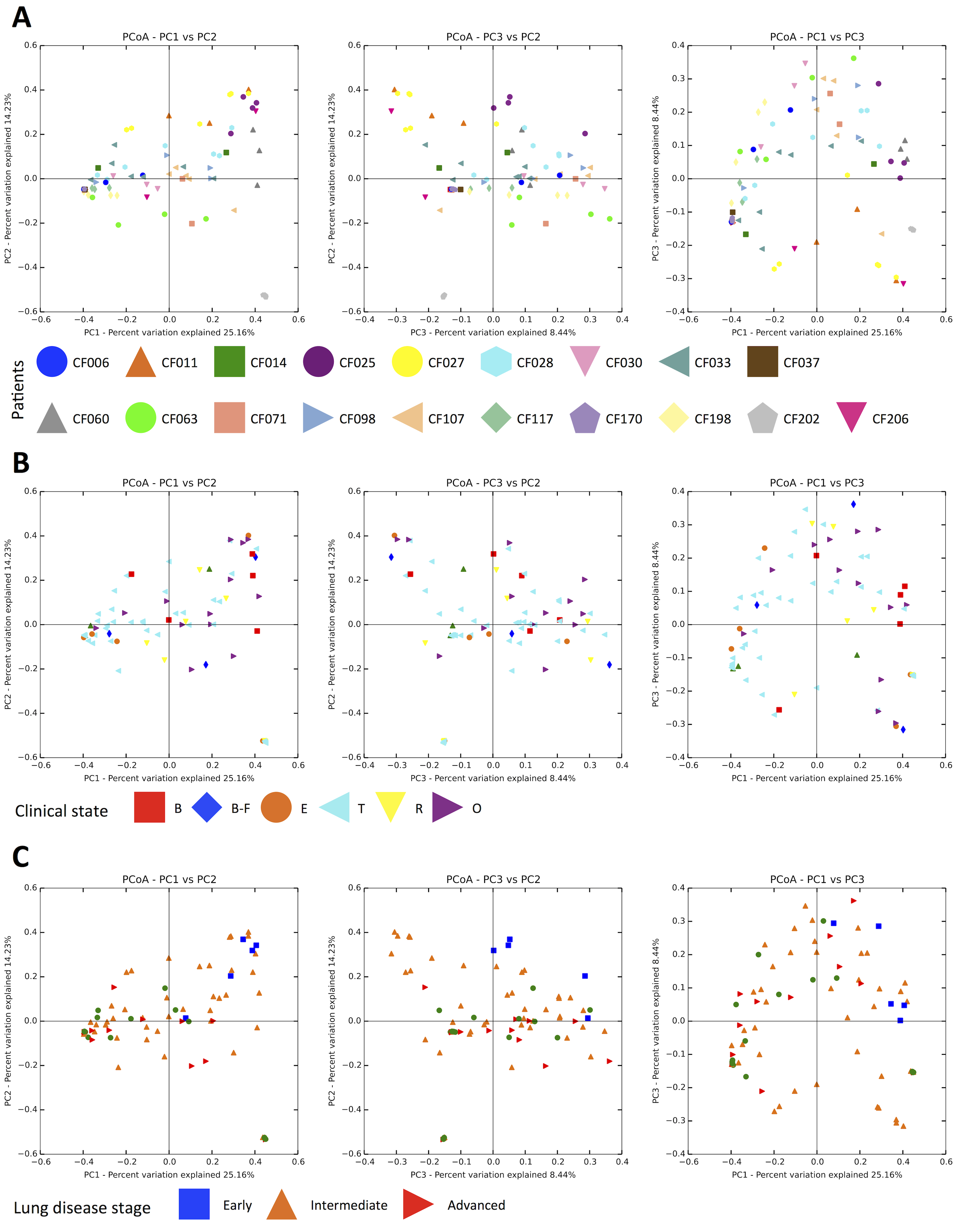

Supplement: S6 Fig — (A) Patient ID. (B) Clinical state. B: baseline–well or mild increase in pulmonary symptoms; not a doctor defined pulmonary exacerbation; not hospitalized for increase in pulmonary symptoms; not on episodic antibiotics for > 30 days; may or may not be on maintenance antibiotics; E: exacerbation–doctor defined pulmonary exacerbation or increased pulmonary symptoms fitting definition of exacerbation; before start of episodic IV or oral antibiotics; not on episodic antibiotics for >30 days; may or may not be on maintenance antibiotics; T: treatment–on IV or oral episodic antibiotics for treatment of doctor defined exacerbation or increased pulmonary symptoms fitting definition of exacerbation; R: recovering–off episodic antibiotics < 30 days; may or may not be on maintenance antibiotics; may or may not be back to baseline clinical state; O: other–patient is on antibiotic therapy for something other than a pulmonary exacerbation; B-F: baseline, but drop in lung function–clinic notes indicate drop in lung function, but no antibiotic prescription was given that day for treatment or other complications. (C) Lung disease stage based on FEV % prediction. Early: >70; Intermediate: between 70 to 40; Advanced: <40. (TIFF) [file ppat.1005308.s006.tiff]

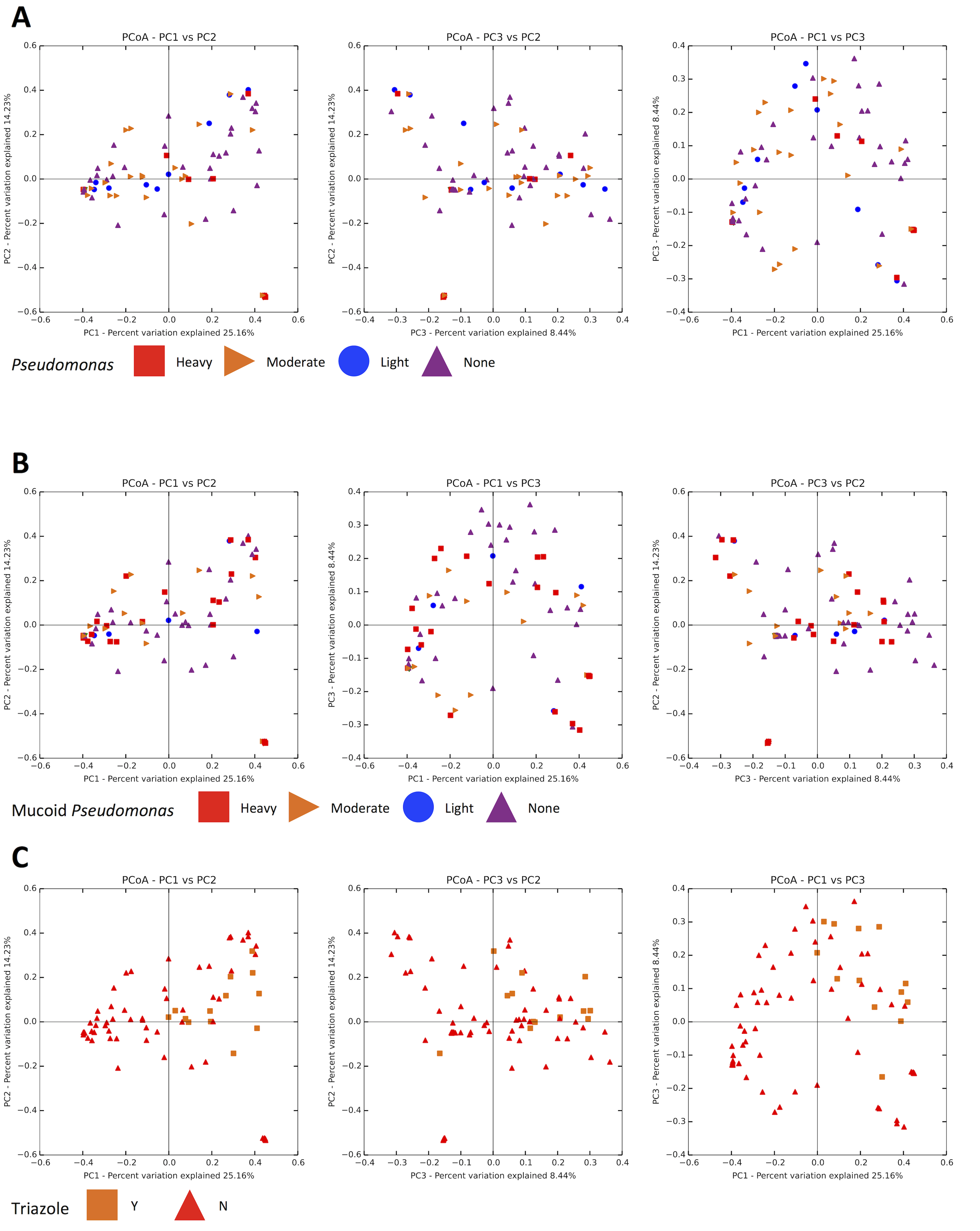

Supplement: S7 Fig — (A) Pseudomonas load. (B) Mucoid Pseudomonas load. (C) Triazole prescription. (TIFF) [file ppat.1005308.s007.tiff]

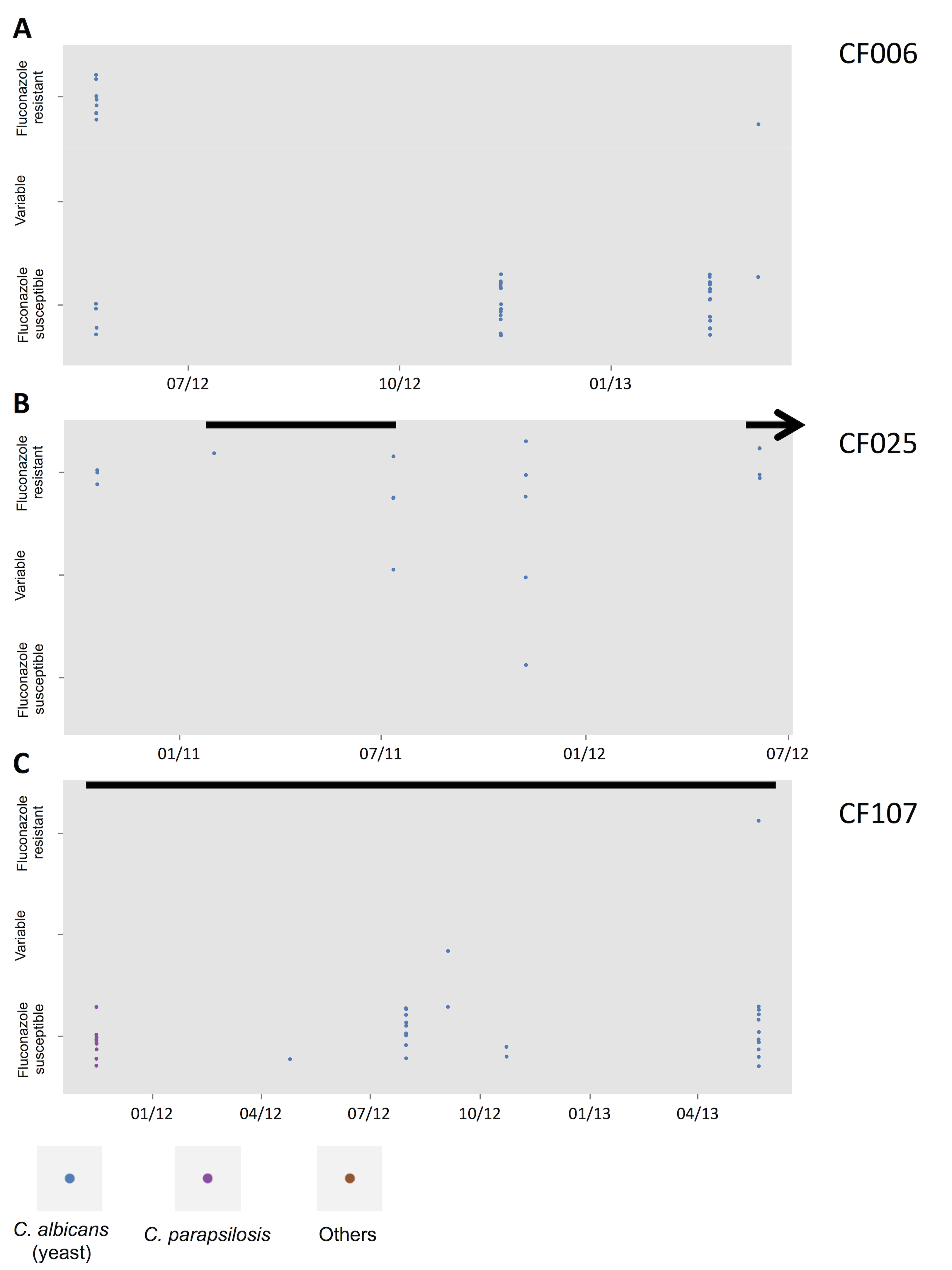

Supplement: S8 Fig — Each dot represents a yeast isolate from the corresponding cystic fibrosis patient and colours represent species identity. The vertical dispersion of isolates within each phenotypic class is simply for visual clarity, as with the horizontal dispersion of isolates from an individual patient. The black line indicates the duration of azole treatment. (A) Summary of isolates from patient 006. (B) Summary of isolates from patient 025. (C) Summary of isolates from patient 107. The isolates are categorized as: “Fluconazole resistant” if their relative growth with fixed concentration of fluconzaole at 128 μg/ml was greater than 2 times that of the relative growth of reference C. albicans strain SN95; and “Fluconazole susceptible” if their relative growth was less than 2 times the relative growth of SN95. (TIFF) [file ppat.1005308.s008.tiff]

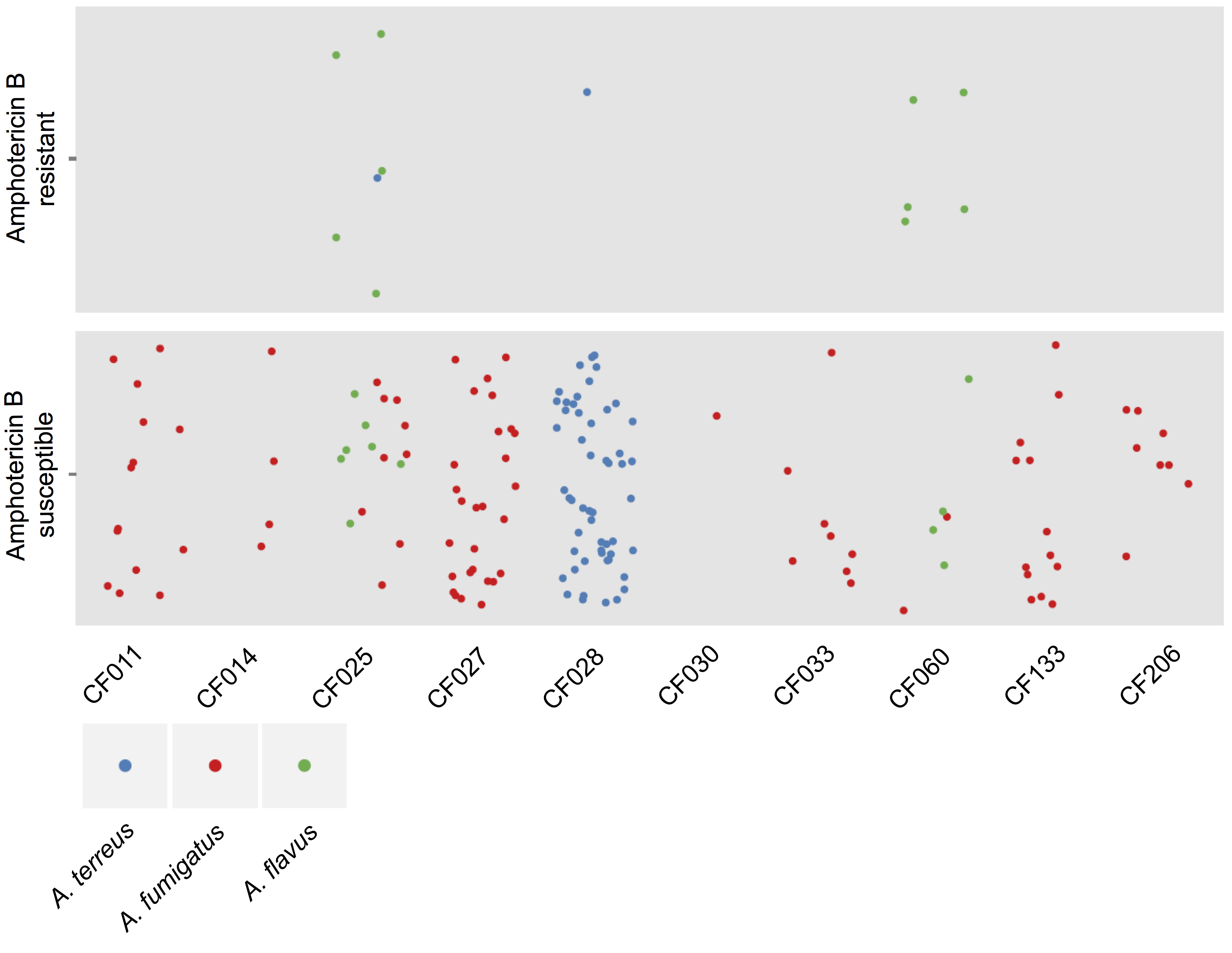

Supplement: S9 Fig — Each dot represents a mold isolate from the corresponding cystic fibrosis patient and colours represent species identity. The isolates are categorized as: “Amphotericin B resistant” if their relative growth with fixed concentration of amphotericin B at 1 μg/ml was greater than 20% of the no drug control; and “Amphotericin B susceptible” if their relative growth was less than 20% of the no drug control. The vertical dispersion of isolates within each phenotypic class is simply for visual clarity, as with the horizontal dispersion of isolates from an individual patient. (TIFF) [file ppat.1005308.s009.tiff]

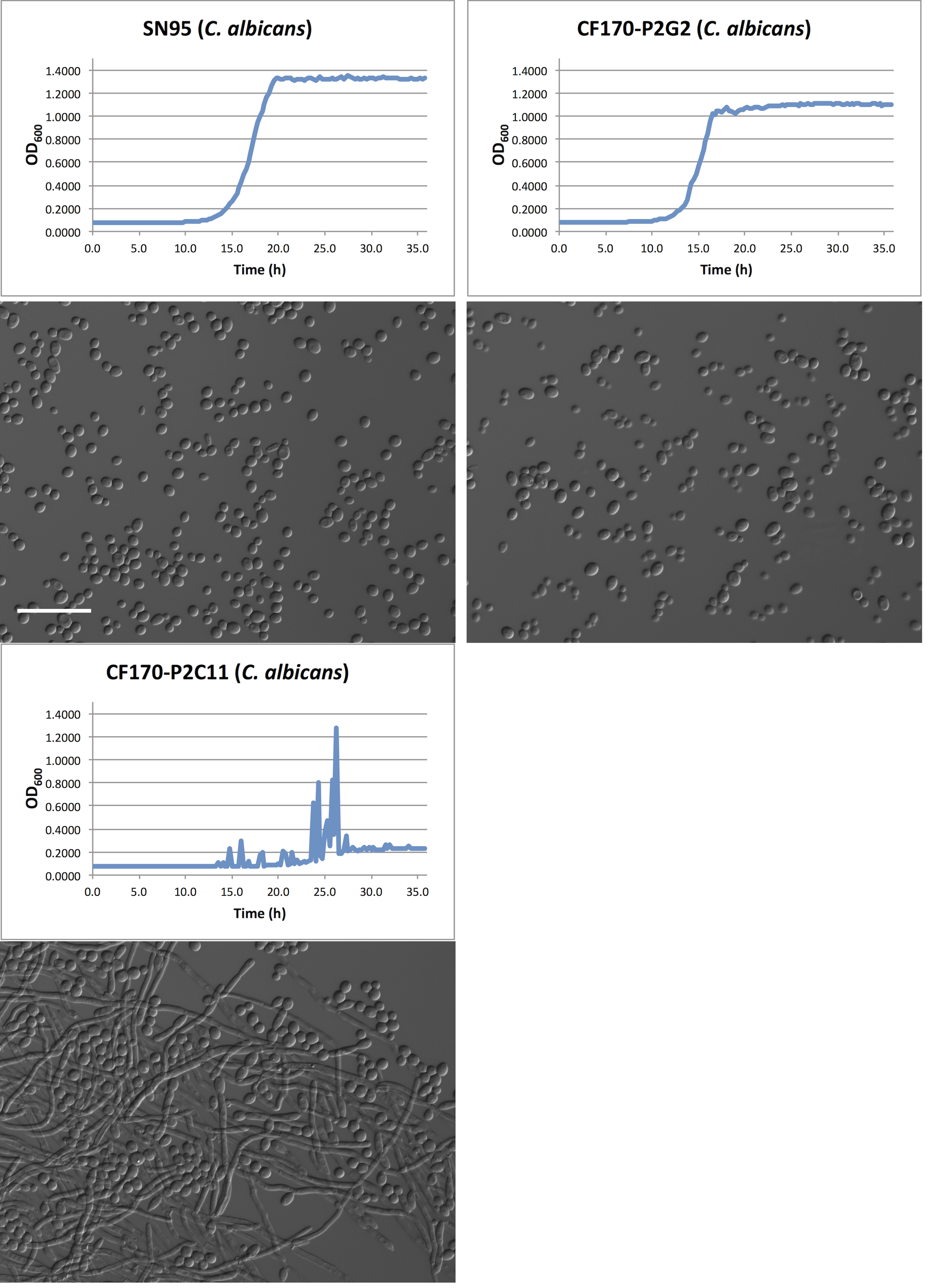

Supplement: S10 Fig — Kinetic measurements of OD600 and DIC images of the reference strain and representative clinical isolates. Scale bar represents 50 μm. (TIFF) [file ppat.1005308.s010.tiff]

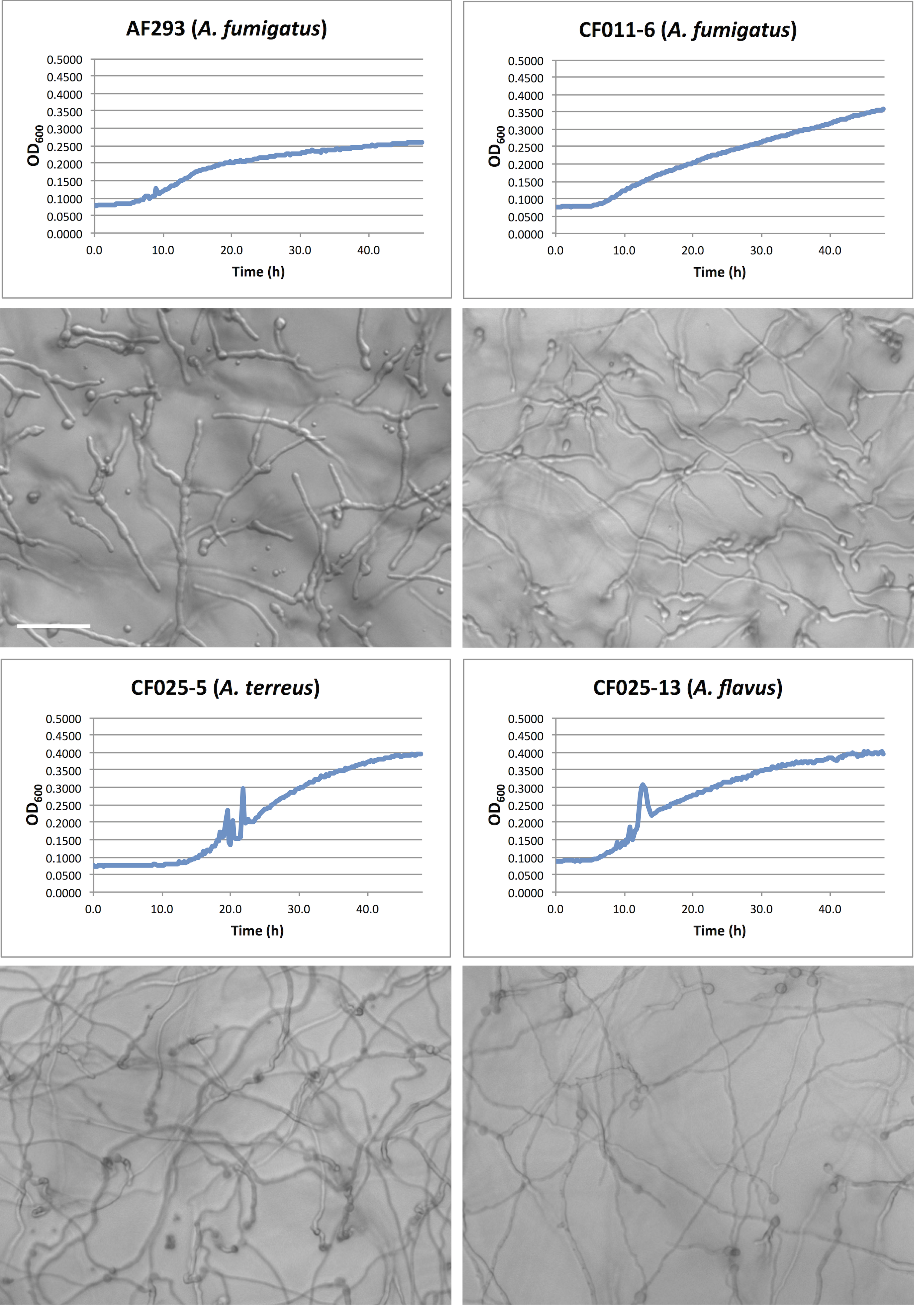

Supplement: S11 Fig — Kinetic measurements of OD600 and DIC images of the reference isolate and representative clinical isolates. Scale bar represents 50 μm. (TIFF) [file ppat.1005308.s011.tiff]

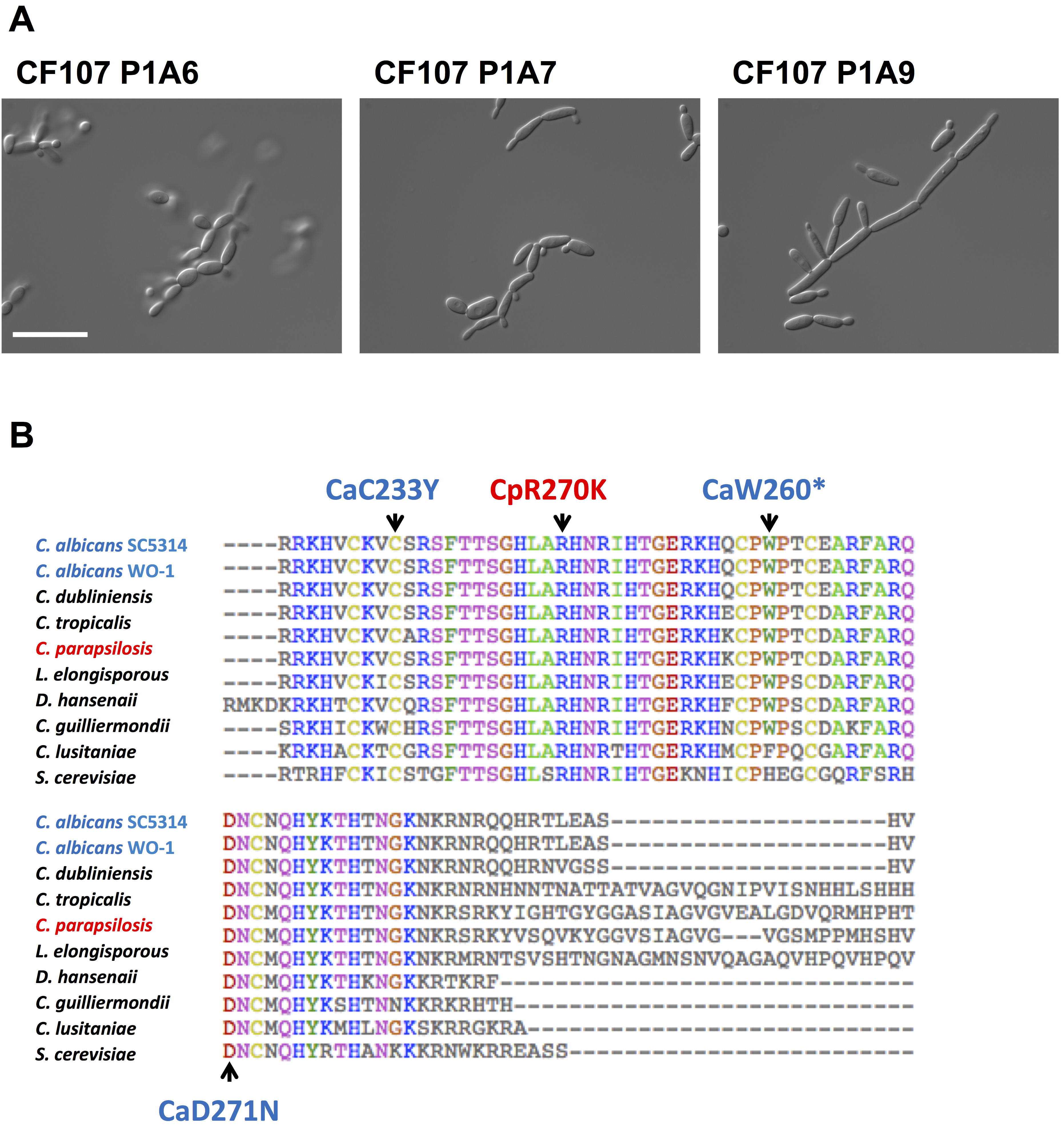

Supplement: S12 Fig — (A) DIC microscopy images of three filamentous C. parapsilosis isolates. Scale bar represents 20 μm. (B) MUSCLE alignment of the C2H2 DNA binding region of NRG1 from closely related yeast species. Arrows indicate the mutations identified from filamentous C. albicans and C. parapsilosis isolates. (TIFF) [file ppat.1005308.s012.tiff]

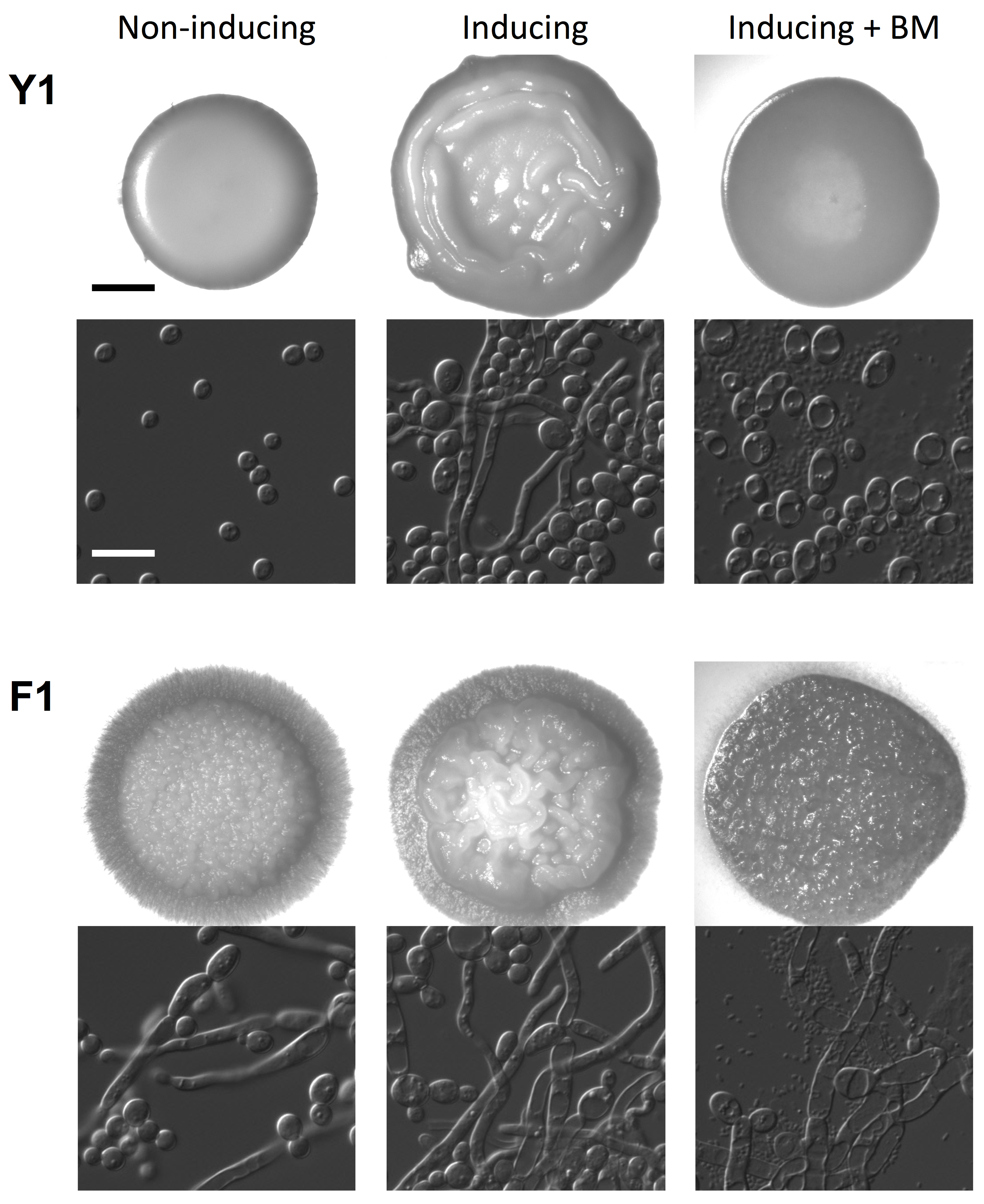

Supplement: S13 Fig — The non-inducing condition is synthetic defined (SD) medium at 30°C and the inducing condition is SD + 5 mM N-acetylglucosamine at 37°C for 48 hours. For the B. multivorans (BM) treatment, 100 μl of ATCC17616 B. multivorans overnight culture was included. Images of colonies are provided in the first and third rows, and DIC microscopy images of cells from the colony are in second and fourth rows. Scale bar on spot image represents 2 mm, and scale bar on DIC image represents 50 μm. (TIFF) [file ppat.1005308.s013.tiff]

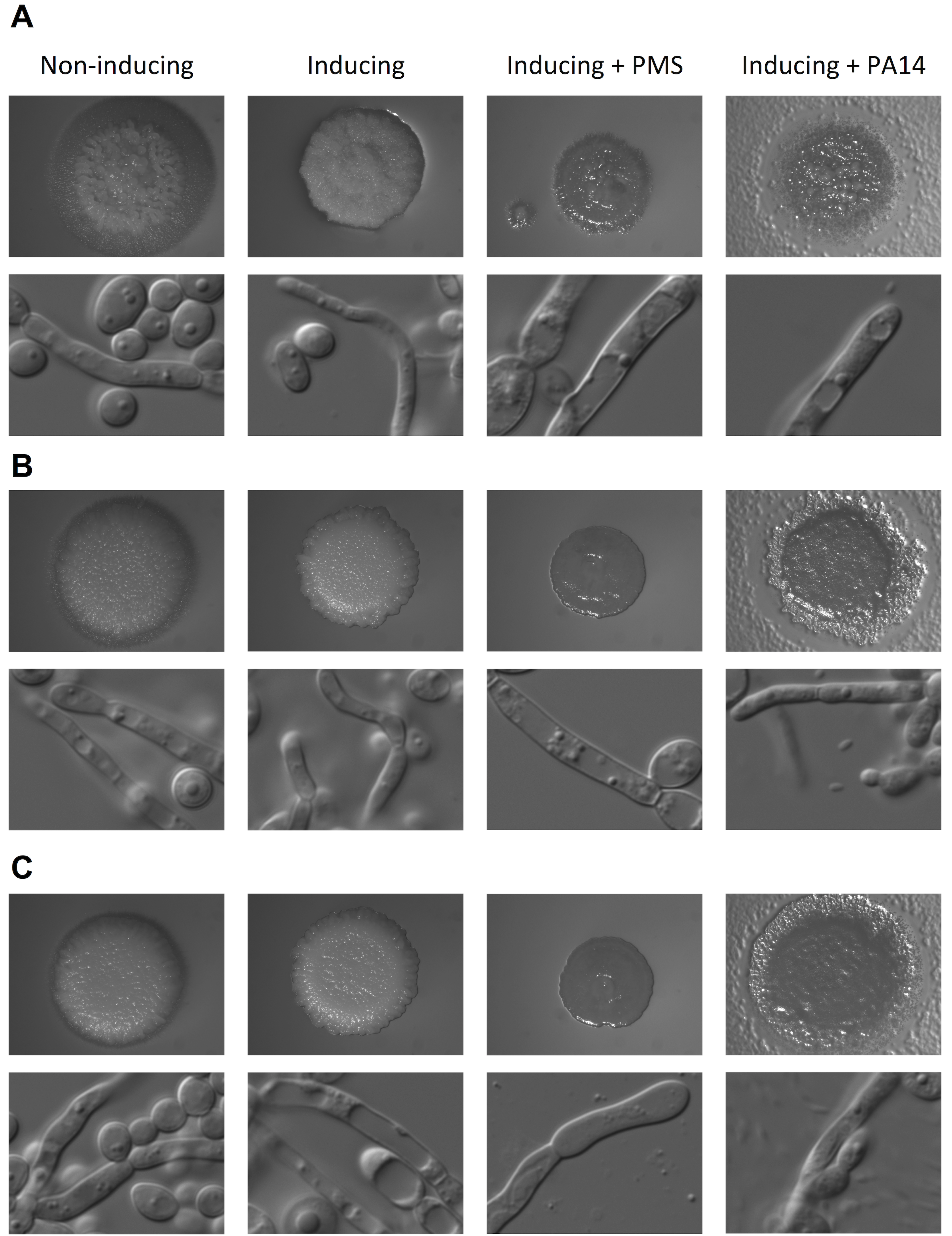

Supplement: S14 Fig — The non-inducing condition is synthetic defined (SD) medium at 30°C and the inducing condition is SD + 5 mM N-acetylglucosamine at 37°C for 48 hours. For the PMS treatment, 5 μM PMS was included, and for the PA14 treatment, 100 μl of PA14 P. aeruginosa overnight culture was included. Images of colonies are provided in the first and third rows, and DIC microscopy images of cells from the colony are in second and fourth rows. Scale bar on spot image represents 2 mm, and scale bar on DIC image represents 10 μm. Representative filamentous isolates from three different patients are shown. (A) CF028 isolate NP1B6 (Q118* in NRG1). (B) CF033 isolate NP1E3 (Y138* in NRG1). (C) CF066 isolate NP1E12 (C233Y in NRG1). (TIFF) [file ppat.1005308.s014.tiff]

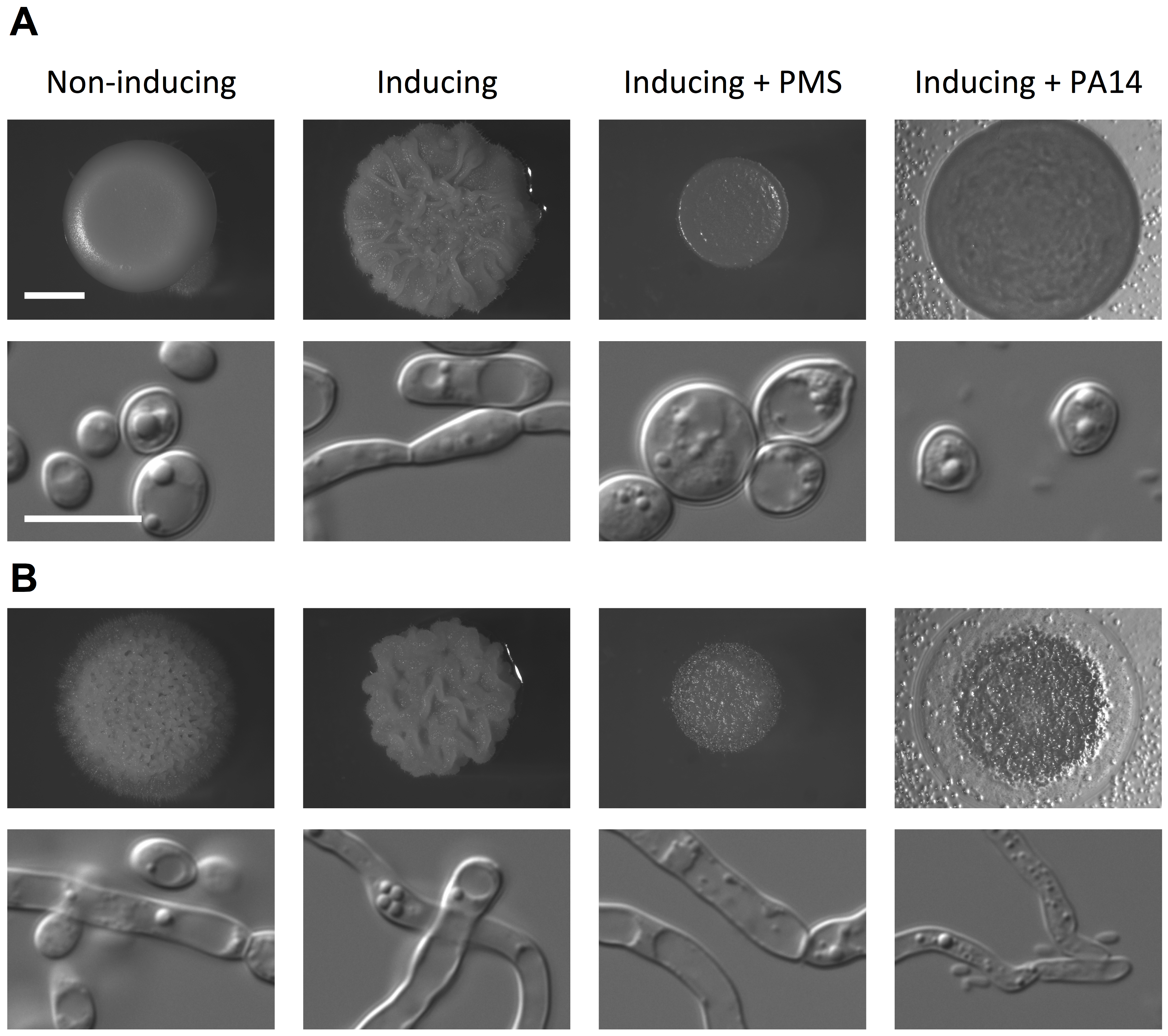

Supplement: S15 Fig — The non-inducing condition is synthetic defined (SD) medium at 30°C and the inducing condition is SD + 5 mM N-acetylglucosamine at 37°C for 48 hours. For the PMS treatment, 5 μM PMS was included, and for the PA14 treatment, 100 μl of PA14 P. aeruginosa overnight culture was included. Images of colonies are provided in the first and third rows, and DIC microscopy images of cells from the colony are in the second and fourth rows. Scale bar on spot image represents 2 mm, and scale bar on DIC image represents 10 μm. (A) Wild-type parental strain. (B) nrg1Δ/nrg1Δ deletion mutant. (TIFF) [file ppat.1005308.s015.tiff]
